# Transphonologization of onset voicing: revisiting Northern and Eastern Kmhmu’

**DOI:** 10.1515/phon-2022-0029

**Published:** 2022-12-16

**Authors:** James Kirby, Pittayawat Pittayaporn, Marc Brunelle

**Affiliations:** Institute of Phonetics and Speech Processing, Ludwig-Maximilians-Universität München, Munich, Germany; Department of Linguistics & Southeast Asian Linguistics Research Unit, Chulalongkorn University, Bangkok, Thailand; Department of Linguistics, University of Ottawa, Ottawa, Canada

**Keywords:** Kmhmu’, phonation, register, tone

## Abstract

Phonation and vowel quality are often thought to play a vital role at the initial stage of tonogenesis. This paper investigates the production of voicing and tones in a tonal Northern Kmhmu’ dialect spoken in Nan Province, Thailand, and a non-tonal Eastern Kmhmu’ dialect spoken in Vientiane, Laos, from both acoustic and electroglottographic perspectives. Large and consistent VOT differences between voiced and voiceless stops are preserved in Eastern Kmhmu’, but are not found in Northern Kmhmu’, consistent with previous reports. With respect to pitch, f0 is clearly a secondary property of the voicing contrast in Eastern Kmhmu’, but unquestionably the primary contrastive property in Northern Kmhmu’. Crucially, no evidence is found to suggest that either phonation type or formant differences act as significant cues to voicing in Eastern Kmhmu’ or tones in Northern Kmhmu’. These results suggests that voicing contrasts can also be transphonologized directly into f0-based contrasts, skipping a registral stage based primarily on phonation and/or vowel quality.

## 1 Introduction

### 1.1 Background

In many of the world’s languages, especially in Asia, the neutralization of a voicing contrast in onsets results in the development of a two-way contrast in pitch, i.e., *tone*, or in a bundle of acoustic properties including pitch, but also duration, phonation, and formant modulations, i.e., *register*. The boundary between tone and register is famously fuzzy (Abramson and Luangthongkum 2009), and it is probably more appropriate to place them on a continuum of ‘tonation’ (Bradley 1982).

Since the discovery of the diachronic relation between voicing and tonation, several models have proposed that the outcome of the transphonologization of voicing is predictable. Haudricourt (1965) proposed that the neutralization of voicing results in a two-way split of the tone system in previously tonal languages but in registrogenesis in atonal languages. Subsequently, authors such as Thurgood (2002: 357) have argued that phonation is effectively an obligatory factor in tonogenesis and that a stage at which phonation type is distinctive in the emergence of tone and register has occurred “in most, if not all cases”.

However, there is evidence that these mechanistic scenarios may need to be revisited. While there is good evidence that the emergence of contrastive tone or register *can* be mediated by onset-conditioned breathy phonation, at the very least in Sino-Tibetan (Cao and Maddieson 1992; Mazaudon 2012; Mazaudon and Michaud 2008; Shi 2020; Watters 2002), studies of languages phonologizing f0 outside of Southeast Asia have not found a significant phonation component (Coetzee et al. 2018; Howe 2017). Along the same lines, some Palaungic and Tibetan varieties with two-way tone systems diachronically traceable to a voicing contrast show no remnants of phonation (Conver 1999 on Lamet; Sun 2003 on Tibetan dialects; Svantesson 1989 on Blang). Particularly important is Kmhmu’ [kjg], also spelled as Kammu or Khmu in the literature, which comprises several closely related dialects spoken by approximately 800,000 speakers in northwestern Laos and across the borders in Thailand, China, and Vietnam (Premsrirat 2002). A member of the Khmuic branch of the Austroasiatic family (Sidwell 2015), Kmhmu’ is a rare case of a language that attests a range of dialects at different stages of the transphonologization process. While some conservative dialects preserve a voicing contrast, others are claimed to have transphonologized voicing into tone or register (Abramson et al. 2007; Lindell et al. 1980, 1981; Ong-arj 1988; Premsrirat 2001, 2002, 2004; Svantesson 1983; Svantesson and House 2006).

In this paper, we explore the role that phonation plays in tonogenesis by looking at the phonetic properties of two Kmhmu’ dialects that have different laryngeal contrasts – one that still preserves a voicing distinction and another that has developed a tonal contrast. Building upon the pioneering phonetic studies of Gårding and Lindell (1978), Svantesson and House (2006), and Abramson et al. (2007), our research investigates the production of voicing and tones from both acoustic and electroglottographic perspectives based on a larger set of words and a greater number of speakers. Our main goal is to determine if there is evidence for incipient or redundant vowel quality or phonation in their voicing and tone contrasts.

### 1.2 Kmhmu’ dialects and laryngeal contrasts

Kmhmu’ occupies a special place in the research on transphonologization of laryngeal contrasts into prosodic distinctions thanks to its dialectal variation that attests different stages of the transphonologization of onset voicing. Previous research (Premsrirat 2001, 2004; Svantesson and House 2006) has demonstrated that many Kmhmu’ dialects have developed a purely f0-based contrast out of an original voicing contrast in onsets. The Proto-Kmhmu’ sound system is believed to have had a contrast between voiced and voiceless consonants very similar if not identical to that of dialects spoken in the eastern part of the Kmhmu’-speaking area (Premsrirat 2001; Svantesson and Holmer 2014), illustrated in Table 1. In addition to the voicing contrast that divided not only stops but also sonorants into voiced and voiceless series, pre-glottalized consonants are set apart as a third distinctive series.1The pre-glottalized phonemes could have been realized as nasals or stops. The correspondences between Western Kmhmu’ ˀm- and ˀn- to Eastern Kmhmu’ ˀm-/ˀb- and ˀn-/ˀd- suggests either *ˀm- and *ˀn-, on one hand, or *ˀb- and ˀd- on the other hand.


**Table 1:** Comparison of Kmhmu’ dialects representing stages of tonogenesis (modified from Premsrirat 2004: 14–15).

Gloss	Stage I (Eastern: Ou, Am, Cwang)	Stage II (Northern: Lue, Krong)	Stage III	
			*b > pʰ (Western: Rok)	*b > p (Northern: Kwaen)
‘to fart’	[pûːm]	[pûːm]	[púːm]	[pûːm]
‘to chew’	[bùːm]	[pṳ̀ːm]	[pʰùːm]	[pùːm]
‘to take a bite’	[pók]	[pók]	[pók]	[pók]
‘to cut down a tree’	[bòk]	[pò̤k]	[pʰòk]	[pòk]
‘astringent’	[câŋ]	[câŋ]	[cáŋ]	[câŋ]
‘to weigh’	[ɟàŋ]	[cà̤ŋ]	[cʰàŋ]	[càŋ]
‘eagle’	[klâːŋ]	[klâːŋ]	[kláːŋ]	[klâːŋ]
‘stone’	[ɡlàːŋ]	[klà̤ːŋ]	[kʰlàːŋ]	[klàːŋ]
‘paddy rice’	[ʰŋɔ́ʔ]	[ŋɔ́ʔ]	[ŋɔ́ʔ]	[ŋɔ́ʔ]
‘to fear’	[ŋɔ̀ʔ]	[ŋɔ̤̀ʔ]	[ŋɔ̀ʔ]	[ŋɔ̀ʔ]
‘monkey’	[ʰwáʔ]	[wáʔ]	[wáʔ]	[wáʔ]
‘to chase’	[wàʔ]	[wà̤ʔ]	[wàʔ]	[wàʔ]
‘tooth’	[ʰrâːŋ]	[râːŋ]	[ráːŋ]	[râːŋ]
‘flower’	[ràːŋ]	[rà̤ːŋ]	[ràːŋ]	[ràːŋ]

**Figure 1:**
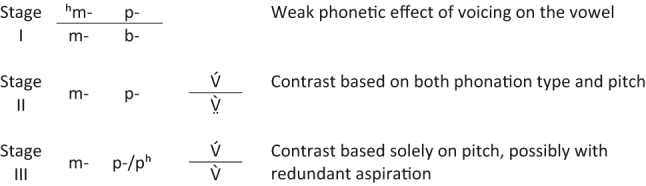
Schematized stages of Kmhmu’ tonogenesis after Premsrirat (2001).

In accordance with the model first proposed by Haudricourt (1961, 1965, when the voicing contrast was lost in now tonal Kmhmu’ dialects, Proto-Kmhmu’ voiced initial consonants should have conditioned phonetic differences in phonation on the following vowel, which would subsequently have evolved into a simple binary register distinction (Ferlus 1979; Premsrirat 2001; Svantesson 1989). This scenario is schematized in Figure 1. However, the reality of a stage in which pitch and phonation on the vowel co-exist as cues to the tonation contrast is debatable. While some authors (Ferlus 1979; Haudricourt 1965; Michaud and Sands 2020; Svantesson and House 2006; Thurgood 2002) propose that contrastive phonation is a crucial element of tonogenesis and/or suggest that it is phonation modulations that initially condition the development of a pitch contrast, recent studies of tonogenesis from voicing neutralization in Afrikaans (Coetzee et al. 2018) and Malagasy (Howe 2017) suggest that it is possible to transphonologize voicing into a pitch-based tonal contrast without going through a phonation stage (see also Gehrmann 2022).

The credibility of the register-based model of tonogenesis comes from reported phonological diversity among present-day Kmhmu’ dialects, which Svantesson and House (2006) classify into three major groups.2The classification of Kmhmu’ by Lindell et al. (1980, 1981 posits two dialect groups, namely Southern and Northern. Subsequent work (Premsrirat 1987 et seq.; Svantesson 1983; Svantesson and House 2006) renamed the Southern group as Eastern, in addition to recognizing a separate Western group. The Northern and the Western groups differ with respect to their reflexes of voiced stops, which are aspirated in the Western Kmhmu’, but unaspirated in the Northern Kmhmu’. The first is Eastern Kmhmu’, which is reported to preserve the historical voicing distinction without accompanying phonation or pitch modulations. Also known as Ou or Cwang, it includes the sub-dialects spoken in Hua Phan province (Laos) and Kim Hoa village (Nghệ An, Vietnam), to cite a few. These varieties all lack a tonal distinction, remaining at Stage I (Premsrirat 2004). Noteworthy is the puzzling case of the variety spoken in Pung Soa village (Yunnan, China), located in the northern part of the Kmhmu’-speaking area. According to Premsrirat (2001) and Svantesson and House (2006), it should be classified as an Eastern Kmhmu’ sub-dialect due to its preservation of the voicing distinction. It also displays the negator /pəː/, which Lindell et al. (1980, 1981 take to be a characteristic of the Yuan sub-dialect of Northern Kmhmu’. Even if one accepts that a single morpheme can be a reliable diagnostic feature, this discrepancy suggests to us that Eastern Kmhmu’ varieties do not form a unified dialect, especially given that retention of the voicing contrast is not a shared innovation, which in turn suggests that Kmhmu’ tonogenesis is a relatively late innovation that diffused across dialect boundaries.

The second and third dialect groups recognized by Svantesson and House (2006) are referred to as “Northern Kmhmu’,” and “Western Kmhmu’,” respectively.3
Premsrirat (2004) put these two together in one branch that she calls “Western Kmhmu’.” These dialects have devoiced the original voiced onsets and developed a true tonal contrast. In both groups, voiced stops *b-, *d-, *ɟ-, and *ɡ- are absent because they have all become either plain voiceless /p-/, /t-/, /c-/, and /k-/ in the former, or aspirated /pʰ-/, /tʰ-/, /cʰ-/, and /kʰ-/ in the latter (Svantesson and House 2006). Crucially, both dialects developed a high tone in the former voiceless series and a low tone in the former voiced series, and this tonogenesis can be dated back to a few hundred years based on Tai loanwords (Svantesson 2011). Based on a geographically comprehensive survey by Premsrirat (2002), the Northern varieties may be further characterized as having either “register complexes” combining pitch and phonation, e.g. the Lue sub-dialect spoken in Nalae village (Oudomxay, Laos), and the Krong sub-dialect spoken in Huay Yen village (Chiang Rai, Thailand), or “pure tones”, e.g. the Kwaen sub-dialect in Om Kae village (Yunnan, China). These two types would represent Stages II and III in the tonogenetic model in Figure 1, respectively. The Western varieties, on the other hand, seem to all rely solely on pitch, e.g. the Rawk sub-dialect spoken in Phon Kaew (Oudomxay, Laos), representing Stage III of tonogenesis. This dialectal diversity provides a natural laboratory in which to study the transphonologization of laryngeal contrast into tone and register.

Although viewing non-tonal, registral, and purely tonal varieties as attestations of sequential stages of tonogenetic development offers a parsimonious explanation of prosodic diversity in Kmhmu’, one puzzle is the absence of dialects that represent an intermediate stage between Stage I and Stage II, in which onset voicing and salient phonation and pitch modulations on the following vowel coexist. Another unsettling gap is the lack of instrumental descriptions of Stage II Kmhmu’ varieties with a register contrast based primarily on phonation and/or vowel quality. The only instrumental study of a dialect impressionistically judged to be registral, namely the Rawk sub-dialect spoken in Huay Steng village (Nan, Thailand), only found positive evidence for (redundant) voice quality differences in female speakers, and concluded that the variety had evolved into a register system based almost exclusively on pitch (Abramson et al. 2007).

While phonation differences are still understudied, the role of f0 in the voicing contrast of Eastern Kmhmu’ (Stage I) and the tone contrast of non-registral Western and Northern Kmhmu’ varieties (Stage III) is fairly well-described. In terms of production, speakers of the former have a slightly higher average f0 after voiceless onsets than after voiced ones (Svantesson and House 2006). On the other hand, speakers of the latter show a significant difference between the average f0 of words corresponding to voiced and voiceless in Eastern Kmhmu’ (Abramson et al. 2007; Gårding and Lindell 1978; Svantesson and House 2006). Perceptually speaking, speakers of the conservative dialects fail to distinguish between pairs of words that differ only with respect to f0, while speakers of the innovative varieties are extremely sensitive to small f0 differences, which they were able to exploit in stimulus categorization (Abramson et al. 2007; Gårding and Lindell 1978; Svantesson and House 2006). In addition to such categorical differences in f0, the non-tonal and the tonal dialects display additional discrepancies expected from their typological dissimilarities. First, the conservative Eastern Kmhmu’ displays a wider pitch range compared to the more innovative Northern Kmhmu’ (Karlsson et al. 2007). Moreover, intonational effects are more limited in the tonal Northern Kmhmu’ in comparison to the non-tonal Eastern Kmhmu’ (Karlsson et al. 2012). These phonetic differences clearly point to a tonal distinction that is present in Northern Kmhmu’ but absent in Eastern Kmhmu’.

Intriguingly, while pitch has been shown beyond doubt to be contrastive in certain Northern and Western Kmhmu’ varieties, no instrumental study has uncovered significant registral properties like phonation and vowel quality in the modern reflexes of the Proto-Kmhmu’ voicing contrast, even at a subphonemic level. This absence raises the possibility that phonation need not be involved in the early development of tonal contrasts and suggests that purely tonal Kmhmu’ dialects may not have gone through a stage in which tones were conditioned by phonation.

### 1.3 The transphonologization of voicing into tone and register

As mentioned in the introduction, the transphonologization of a voicing contrast in onset obstruents, and sometimes in onset sonorants, is a typologically common diachronic phenomenon. Well-attested outcomes of this process include the emergence of a pitch-based contrast between a high and a low tone (Haudricourt 1954; Hyman 1976; Matisoff 1973), or a more complex multidimensional contrast involving pitch, phonation and vowel quality called *register* (Brunelle and Tấn 2021; Ferlus 1979; Henderson 1952; Huffman 1976). In languages that have exclusively pitch-based tone systems, a high pitch is associated with former voiceless stops while a low pitch is the reflex of former voiced stops. In register systems, the voiced series normally leads to the development of a low register associated with a breathy phonation, closed vowels or falling diphthongs and a lower pitch while the voiceless series is replaced with a high register that has a modal voice quality, more open vowels and rising diphthongs and a higher pitch. The unfolding of tonogenesis and registrogenesis was either inferred (Hyman 1976) or reconstructed based on a comparison of languages at apparently different stages of the process (Ferlus 1979; Huffman 1976), but it is only recently that fine-grained phonetic experiments have been able to begin studying real-time variation and change in laryngeal contrasts (Brunelle et al. 2022; Coetzee et al. 2018; Howe 2017).

The phonetic properties associated with tone and register are normally assumed to be traceable to secondary properties of voicing. The best-described is the secondary role of f0, that seems systematically higher at the beginning of vowels following voiceless than voiced obstruents, regardless of other phonetic aspects of the realization of the voicing contrast (Dmitrieva et al. 2015; Hanson 2009; House and Fairbanks 1953; Kingston and Diehl 1994; Kirby and Ladd 2016; Lisker 1986; Ohde 1984; Rousselot 1897 and many others). Other properties that have been associated with phonetic voicing include a lower F1 (Esposito 2002; House and Fairbanks 1953; Stevens and House 1963), a slightly lower F2 (Cole et al. 2010) and a breathier or laxer voice quality (Löfqvist and Mcgowan 1992; Ní Chasaide and Gobl 1993), but these secondary properties appear less salient than f0 and their prevalence is not as firmly established as they are less commonly studied. Furthermore, despite evidence that sonorant voicing can also cause variations in f0 and possibly other phonetic properties, the phonetic mechanisms that account for the secondary properties of obstruent voicing do not obviously extend to sonorants (L-Thongkum 1992). It is therefore not clear if vowel quality and/or phonation type play an indispensable role in tonogenesis. Despite reports that some Kmhmu’ dialects do have registers, some of the purely tonal dialects could have arguably gone directly to the end of the pathway, having skipped the phonation stage, as appears to be the case when f0 phonologizes outside of East and Southeast Asia (Coetzee et al. 2018; Howe 2017).

### 1.4 Research questions

To determine if the transphonologization of voicing into f0 in Kmhmu’ potentially involved a stage where phonation type played a substantive role, we investigated two Kmhmu’ dialects that should be at the beginning and the end of the transphonologization of onset voicing, namely the Eastern Kmhmu’ dialect spoken in and around Vientiane, Luang Prabang, Xiang Khouang, and Bolikhamasay provinces in Laos, and a Northern Kmhmu’ variety spoken in Nan Province, Thailand. While the first dialect is described as preserving the historical proto-Kmhmu’ voicing contrast, the second is spoken near to other dialects described by Premsrirat (1999, 2001 as having register systems based partly or exclusively on f0. These two dialects allow us to expand on the seminal acoustic studies of Kmhmu’ laryngeal contrasts by Svantesson and House (2006) and Abramson et al. (2007) to determine if there are any traces of phonetic properties other than f0 and VOT in conservative and innovative varieties. Such properties could reveal if conservative varieties contain redundant precursors of register alongside with voicing and if innovative dialects contain remnants of former phonation type differences. Our specific research questions are the following:

1) Is the onset voicing contrast still robust in conservative Eastern Kmhmu’? Are there any remnants of the original voicing contrast in this innovative Northern Kmhmu’ variety?
2) Is f0 a robust secondary property of the voicing contrast in Eastern Kmhmu’ and the primary contrastive property of tone in this Northern Kmhmu’ variety?
3) Does the voicing contrast condition differences in phonation and formants in Eastern Kmhmu’? Is the contrast accompanied by differences in phonation and formants in the Northern Kmhmu’ variety?

## 2 Materials and methods

### 2.1 Dialects and participants

The phonologically conservative Eastern Kmhmu’ (hereafter EK) is represented in the present study by the Am (also known as Cwang or Ou) sub-dialect spoken by a majority of Kmhmu’ in and around Vientiane, Luang Prabang, Xiang Khouang, and Bolikhamasay provinces in Lao PDR. Previously described in Osborne (2018) and Kirby (2022), this variety features a relatively large set of consonants with a voicing distinction in both obstruents and sonorants, as shown in Table 2. The voicing contrast is illustrated by such (near-)minimal pairs as /puː/ ‘empty rice husk’ versus /buː/ ‘puffy, swollen’ or /kɔːn/ ‘child’ versus /ɡɔːŋ/ ‘soup’.

**Table 2:** Consonant inventory of Eastern Kmhmu’ (after Osborne 2018).

	Labial	Alveolar	Palatal	Velar	Glottal
Stops	p	t	c	k	ʔ
	pʰ	tʰ	cʰ	kʰ	
	b	d	ɟ	ɡ	
Fricatives		s			h
Nasals	ʰm	ʰn	ʰɲ	ʰŋ	
	ˀm	ˀn	ˀɲ	ŋ	
	m	n	ɲ		
Laterals		ʰl			
		l			
Rhotics		ʰr			
		r			
Glides	ʰw		ʰj		
	w		ˀj		
			j		

On the other hand, the innovative Northern Kmhmu’ (hereafter NK) is represented by the Lue sub-dialect spoken in Huay Lao village, Song Khwae district, in the northeastern corner of Nan Province, Thailand. Much like closely related varieties documented by Premsrirat (2002, 2004, this NK variety has approximately half the number of consonants found in EK, as a voicing contrast is absent in both obstruents and sonorants as illustrated in Table 3 (the labial and alveolar implosives are later innovations, unrelated to the transphonologization process of interest). However, the relatively small consonant inventory is supplemented by a contrast between high and low f0, as illustrated by (near-) minimal pairs like /kɔ́ːn/ ‘child’ versus /kɔ̀ːŋ/ ‘ridge, mountain range’ and /lú:/ ‘k.o. spiced minced meat’ versus /lù:/ ‘to howl (of a dog)’.

**Table 3:** Consonant inventory of Northern Kmhmu’ (Premsrirat 2004).

	Labial	Alveolar	Palatal	Velar	Glottal
Stops	p	t	c	k	ʔ
	pʰ	tʰ	cʰ	kʰ	
	ɓ^a^	ɗ			
Fricatives		s			h
Nasals	m	n	ɲ	ŋ	
Laterals		l			
Rhotics		r			
Glides	w		j		

^a^Implosives are transcribed as preglottalized ˀb and ˀd in Premsrirat (2004).

This study is based on recordings of 42 speakers, 20 EK (12 females and 8 males, ages 21–69 in 2020) and 22 NK (13 females and 9 males, aged 27–67 in 2017). All consultants were native speakers of Kmhmu’ and spoke Kmhmu’ daily as their primary language. They were also fluent in the linguae francae of their respective regions, namely Lao and Northern Thai. In addition, younger NK speakers also spoke Central Thai, the national language of Thailand. The NK recordings were made in Huay Lao Village, Song Khwae Province, Thailand in May 2017. The EK materials, some of which have previously been analyzed in Kirby (2022), were collected in a village outside Vientiane, Lao PDR in January 2020. All audio materials and associated scripts are available as part of this article’s accompanying OSF archive (https://osf.io/wv6qz/).

### 2.2 Materials and procedure

The materials and procedure were designed to be as parallel between the two dialects as possible. For each dialect, speakers were recorded reading a list of words. In EK, each word was produced twice in isolation and twice in a carrier phrase, while in NK it was produced four times in a frame sentence. The carrier phrases used for the two dialects differed slightly. They were /ʔoʔ cə law _____ ʔan klɔh/ “I will say_____ clearly” for the Eastern dialect and /ʔòʔ làw ____ sí mɨ̀ː/ “I say ____ four times” for the Northern dialect. Here, we analyze only the carrier phrase items from the Eastern recordings to facilitate comparison with the Northern recordings.

The word lists were designed with the help of available dictionaries (Premsrirat 2002; Shorto 2006) and checked and adapted with the help of native speakers. We targeted words containing all possible combinations of coronal and velar onsets with the five long vowels /iː ɛː aː ɔː uː/. We selected open monosyllables to the extent possible, but when ideal monosyllables did not exist, sesquisyllables with open final syllables or monosyllables closed by nasal or liquid codas were chosen; in addition, it was occasionally necessary to include words with the vowels /əː oː/ in Eastern Kmhmu’. The final lists for the Eastern and the Northern Kmhmu’ dialects included 59 and 53 words, respectively (see Appendices A 4The full Eastern wordlist included a total of 125 items, of which only a subset is analyzed here. and B).

To facilitate comparison between the dialects, we classify all items as belonging to either low or high register. In Eastern Kmhmu’, this is usually, but not always, the same as voicing, i.e. voiced onsets are classified as low register and voiceless onsets as high register. However, due to the existence of *register spreading* (Brunelle and Tấn 2021), there are a few items with voiceless presyllable onsets (/klaːŋ/ ‘eagle’, /cŋaːr/ ‘yellow’, /pŋaːl/ ‘to warm up slowly’) which belong to the set of high register onsets in spite of the fact that their main syllables onsets are (voiced) sonorants.

Participants produced the Kmhmu’ form in response to an oral prompt of the Lao or Thai gloss by an experimenter. Prior to recording, our research assistants went over the glosses with each participant to make them feel comfortable with the procedure and familiarize them with Kmhmu’ lexical items on the word list. Recordings were made direct to disk using the SpeechRecorder software (Draxler and Jänsch 2004) with a headset condenser microphone. In Vientiane, they were made in a quiet, sound-treated booth, while in Nan, they were conducted in a quiet wooden stilt house. A simultaneous EGG signal was also recorded from most Eastern and all Northern speakers. In Nan, EGG recordings were made using the MATLAB data acquisition toolbox and a Glottal Enterprises EG2-PCX laryngograph connected to a laptop through a National Instrument USB6210 data acquisition device. In Vientiane, EGG data was captured using an EGG-D200 device from Laryngograph Ltd.

#### 2.2.1 Annotation

**Figure 2:**
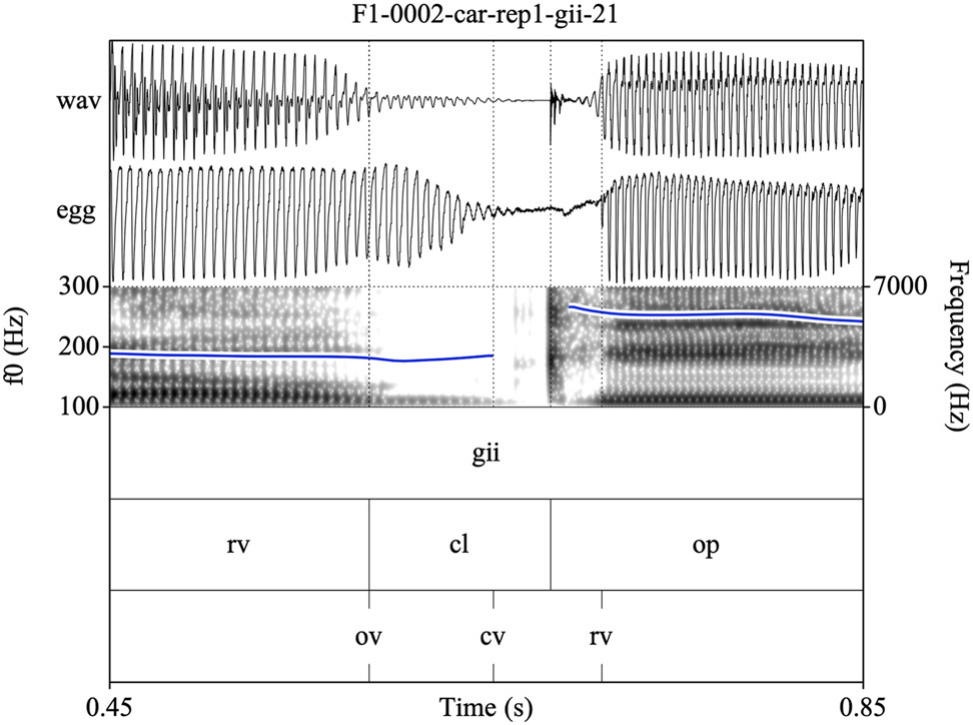
Example of a voiced plosive with partial closure voicing and post-release voice lag for the item /ɡiː/ ‘here, this’, Eastern speaker EF1. EGG signal highpass filtered from 75 Hz. See text for explanation of annotation labels.

**Figure 3:**
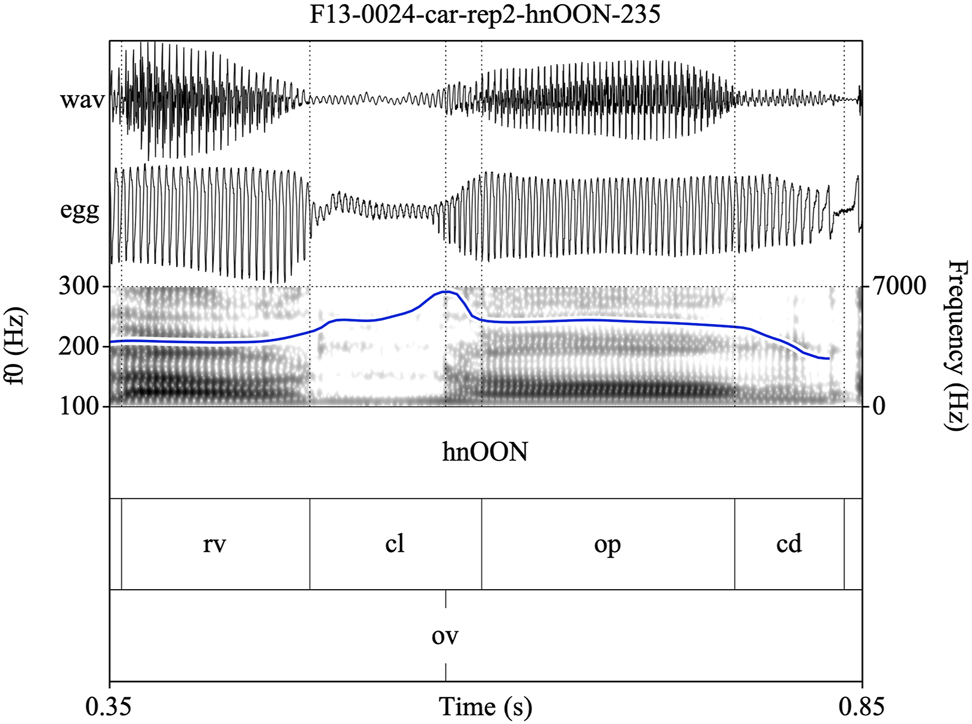
Example of a voiceless nasal with vocal fold vibration throughout the closure phase for the item /ʰnɔːŋ/ ‘still, yet, remain’, Eastern speaker EF13. EGG signal highpass filtered from 75 Hz. See text for explanation of annotation labels.

Target syllables were manually annotated and stored as EMU speech databases (Winkelmann et al. 2017). Annotations were made on three tiers (see Figures 2 and 3). The first tier contained a X-SAMPA transcription of the syllable. The second tier was used to annotate constrictions in the supraglottal vocal tract. The label **cl** was used to delimit the period of oral closure. For plosives, the closure phrase was the period of silence preceding the release burst; for sonorants, this was either the sonorous portion (for voiced nasals or liquids) or a period containing both silence and frication noise followed by a region of periodicity (for voiceless sonorants). The label **op** was used to label the open phase of the following vowel, assessed either as the onset of the plosive release burst (if present) or the onset of periodic formant structure with a clear second formant. If present, a sonorant coda was labeled was **cd**. The label **rv** (‘reference vowel’) was used to indicate the vowel of the preceding item in the carrier phrase. In cases where this item did not immediately precede the closure of interest, the label **ps** (‘preceding segment’) was used to label the temporally preceding segment, so that **rv** always referred to the same reference vowel in all tokens. The third tier was used to mark the onset (**ov**) and possible cessation (**cv**) and subsequent resumption (**rv**) of periodic vocal fold vibration. These were determined with reference to the EGG signal when possible, or with reference to the audiogram for speakers or tokens for which the EGG signal was unreliable. This annotation scheme facilitated measurement of the duration of closure voicing as well as post-release voicing lag within the same syllable.

**Figure 4:**
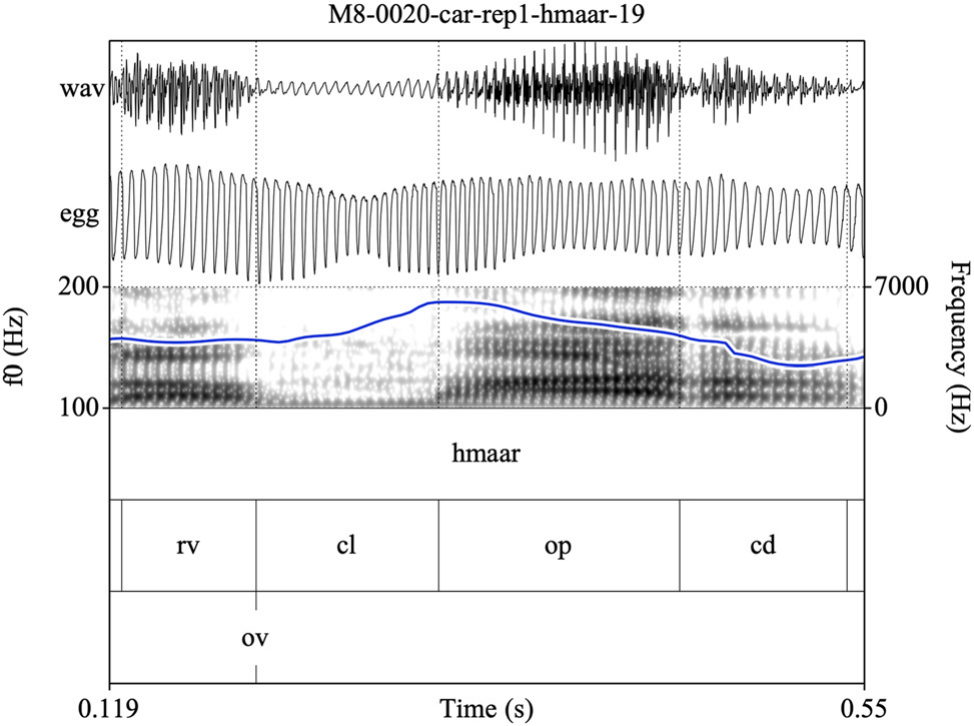
Example of a voiceless nasal with vocal fold vibration throughout the closure phase and no clear indication of velic opening for the item /ʰmaːr/ ‘salt’, Eastern speaker EM8. EGG signal highpass filtered from 75 Hz. See text for explanation of annotation labels.

Annotating sonorants was more challenging. For voiceless nasals, vocal fold vibration would often be indicated throughout the closure phase. In some cases, this would be acoustically distinct from a period of nasal murmur preceding the oral closure release, identifiable by increased waveform amplitude and the presence of formant structure (see Figure 3). However, there were also many examples where determining this instant was difficult or completely arbitrary (see Figure 4). In Section 3.1, we discuss our attempts to distinguish voiced and voiceless sonorants based on acoustic properties of their closure phases.

### 2.3 Acoustic measurements and analysis

In addition to the Voice Onset Time (VOT), we extracted a number of acoustic measurements from the annotated files using PraatSauce (Kirby 2018), a set of Praat scripts for the extraction of spectral measurements based on VoiceSauce (Shue et al. 2011). Acoustic measures were taken at 1 ms intervals over the entire recording. Fundamental frequency (f0) was estimated using Praat’s autocorrelation method within speaker-specific ranges to minimize octave halving/doubling errors. Formant resonances (F1, F2, and F3) were estimated by the Burg LPC algorithm using a ten-pole filter and a 25 ms Gaussian-like analysis window. We used a formant ceiling of 5,000 Hz for male speakers and 5,500 Hz for female speakers, with bandwidths estimated using the formula of Hawks and Miller (1995). Cepstral peak prominence (CPP) was calculated using the method of Hillenbrand et al. (1994) using a lower quefrency of 1/300, parabolic interpolation for peak amplitude detection, and Theil’s robust line fit method.

To resolve spurious tracking errors, all f0, F1, F2, and F3 values were removed when they deviated by more than three standard deviations from the means computed for each combination of subject, vowel, and register. This procedure resulted in the removal of less than 1% of each of the measurement points. Spectral balance measures (H1*–H2*, H1*–A1* and H1*–A3*, corrected using the method of Iseli and Alwan (2006) at timepoints where f0 and F1/F3 had been excluded were also deleted.

Here, we report the fundamental frequency (f0), the first two formants (F1 and F2), cepstral peak prominence (CPP), and the corrected difference between the first two harmonics, H1*–H2*. We focus on these measures as they are frequently found to correlate with phonation type differences in register languages and languages which employ contrastive voice quality (Abramson et al. 2007: 200; Abramson et al. 2015; Brunelle et al. 2020, 2022; DiCanio 2009; Esposito and Khan 2012; Garellek and Keating 2011; Tạ et al. 2022; Watkins 1997 *inter alia*). CPP is an indicator of the regularity of vocal fold vibrations (jitter, shimmer) and of glottal amplitude, both which are both known to be weaker in breathy voice (Fraile and Godino-Llorente 2014; Hillenbrand and Houde 1996). Unlike measures such as H1–H2, computation of CPP does not require estimation of either the fundamental frequency nor the vocal tract resonances, so it provides a useful complement to spectral balances measures.

### 2.4 EGG signal

Given that the relationship between H1*–H2* and the actual glottal open quotient can be highly speaker dependent (Kreiman et al. 2012), we complemented this spectral balance measure with a measure of open quotient derived from EGG. The EGG signals were processed using *praatdet* (Kirby 2017). The low-frequency (Gx) component of the EGG waveform was removed using a high-pass filter with a 40 Hz pass frequency and a 20 Hz smoothing cutoff. Before calculating the glottal duty cycle (open quotient or Oq), Praatdet smooths both the raw electroglottographic (Lx) signal and subsequent derivative (dEGG) transformation by calculating a linearly weighted symmetric moving average over the *k* timepoints preceding and following each period. In this study, *k* = 20.

**Figure 5:**
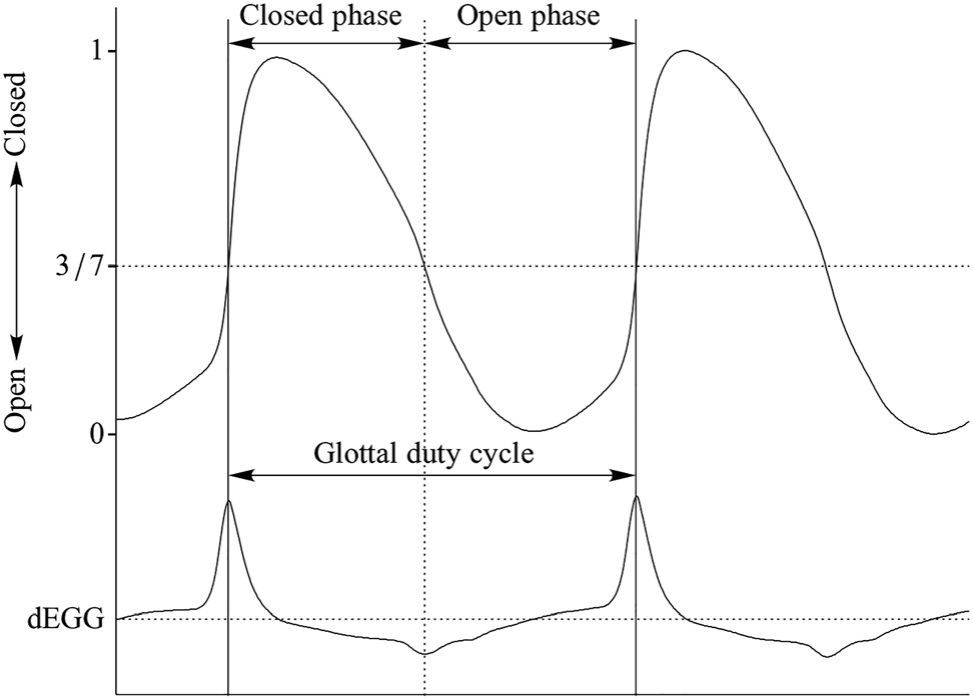
Comparison of Lx waveform (top) and (smoothed) dEGG (bottom). The intersection of the gray lines indicates the point where the negative-going Lx signal crosses an amplitude threshold of 3:7 of the peak-to-peak amplitude. In this example, the chosen amplitude threshold corresponds well to the opening instant as determined from the dEGG signal.

Determining the open quotient from the EGG signal is not always straightforward. While the closing peaks are generally easily identified from the dEGG signal, opening peaks may be indeterminate (Henrich et al. 2004; Michaud 2007). For this reason, while the maximum positive peak in the dEGG is usually used as an indicator the closing instant, an EGG-based threshold method may be used to determine the opening instant. Howard (1995) suggests a point where the negative-going Lx signal crosses an amplitude threshold of 3:7 of that cycle’s peak-to-peak amplitude. The idealized relationship between the opening peak of the dEGG signal and the point on the Lx waveform determined by Howard’s thresholding method is shown in Figure 5. We calculated both the dEGG-based method as well as “Howard’s method”; as the results were not dependent on the method used, we report only the latter here.

After calculating the Oq values for the open phases of our test items, we combined them with the acoustic measures by associating each timepoint at which a spectral measurement (f0, F1…) was taken with the start time of the preceding Lx period. Because acoustic measures were taken every millisecond, any given Lx period is associated with multiple acoustic measures.

### 2.5 Normalization and statistical analyses

To facilitate the comparison of acoustic measurements across participants, spectral measures (f0, formants, spectral amplitudes, and CPP) were converted to speaker-specific *z*-scores. As *z*-scales are less intuitive to interpret, we converted *z*-scores back to original scales based on means and standard deviations for all speakers in each dialect (mean of all speakers + *z*-score * standard deviation of all speakers). These normalized scales are used in figures where data is pooled over groups of speakers.

For each dialect and spectral measure, trajectories were fit to Generalized Additive Mixed Models (GAMMs: Wood 2017; Wieling 2018) in R (R Core Team 2022) using the *mgcv* package (Wood 2017) and helper functions from the *itsadug* package (van Rij et al. 2022). Because we were interested in estimating effects of both register and voicing, and because GAMMs do not straightforwardly handle interactions involving smooth terms, we created custom interaction terms where each level was a combination of Manner, Voicing and Register (e.g. *stop.high.voiceless, rhotic.low.voiced*, etc.) or, for F1 and F2, a combination of Manner, Voicing, Register and Vowel (e.g. *stop.high.voiceless.aa, rhotic.low.voiced.ii*, etc.). We then included this interaction term as a fixed predictor, along with smooth terms for the custom interaction, Vowel (if appropriate), Word, and by-speaker smooths for the interaction term. The relevant R model syntax is shown in (1–2).

(1)

**Table:** 

measure_(f0|H1*-H2*|CPP|CQ_PH)_ ∼ Manner.Reg.Voice +	
	s(times_norm, by = Manner.Reg.Voice) +
	s(times_norm, Vowel, bs="fs", m=1) +
	s(times_norm, Word, bs="fs", m=1) +
	s(times_norm, Speaker, by = Manner.Reg.Voice, bs="fs", m=1)

(2)

**Table:** 

measure_(F1 |F2)_ ∼ Manner.Reg.Voice.Vowel +	
	s(times_norm, by = Manner.Reg.Voice.Vowel) +
	s(times_norm, Word, bs="fs", m=1) +
	s(times_norm, Speaker, by = Manner.Reg.Voice.Vowel, bs="fs", m=1)

Autocorrelation was controlled for with an AR1 model design, defining the starting point for each time series as the onset of the vowel (for f0, F1, F2, H1*–H2* and CPP) or the onset of the open phase (for Oq) and the estimating the autocorrelation parameter *rho* based on the model residuals. As model criticism showed evidence of residuals with extremely heavy tails, all models reported here were fit using a scaled*-t* distribution (family = “scat”).

To provide a general overview of the differences in trajectories, we plot the GAMM model predictions with 95% confidence intervals, but to assess the significance of these estimates, we plot difference smooths between pairs of levels of the interaction term of interest. To give a sense of the magnitude of the effects, we also report differences in the estimated marginal means at vowel onset and midpoint, which were estimated based on the GAMM models using *emmeans* (Lenth 2022). Full model output can be found in Appendix C; data and code are available at https://osf.io/wv6qz/.

## 3 Results

This section reports on the overall acoustic and EGG results of the onsets and the vowels as well as results of individual variation and relative role of acoustic cues.

### 3.1 Onsets

#### 3.1.1 VOT

**Figure 6:**
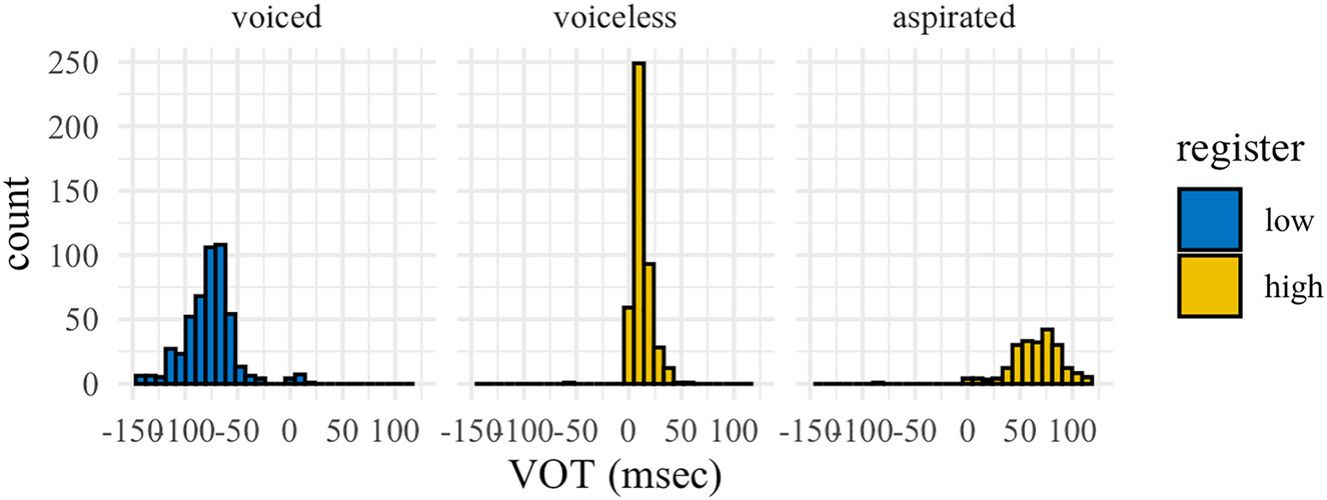
VOT distributions, Eastern Kmhmu’.

Table 4 gives descriptive statistics for VOT and voice lag in Eastern Kmhmu’, plotted in Figure 6. There is a clear lead-lag contrast between low-register (voiced) and high-register (voiceless unaspirated) plosives. Complete devoicing of low-register (voiced) plosives was extremely rare (just 3%, or 17 out of 518 instances), but partial devoicing was more common (around 16%, or 83/518 instances), and 17% (90/518 instances) of voiced plosives were realized with some degree of post-release voice lag, typically in addition to voice lead (as in Figure 2 above).

**Table 4:** VOT and voice lag by voicing and onset articulation (in msec), Eastern Kmhmu’.

Register	Onset	Mean VOT	SD VOT	Mean lag	SD lag
Low	b	−79	29	7	8
	d	−87	41	9	6
	ɡ	−80	29	16	11
High	p	9	5		
	t	10	6		
	k	18	11		
	p^h^	52	24		
	t^h^	59	23		
	k^h^	71	22		

**Figure 7:**
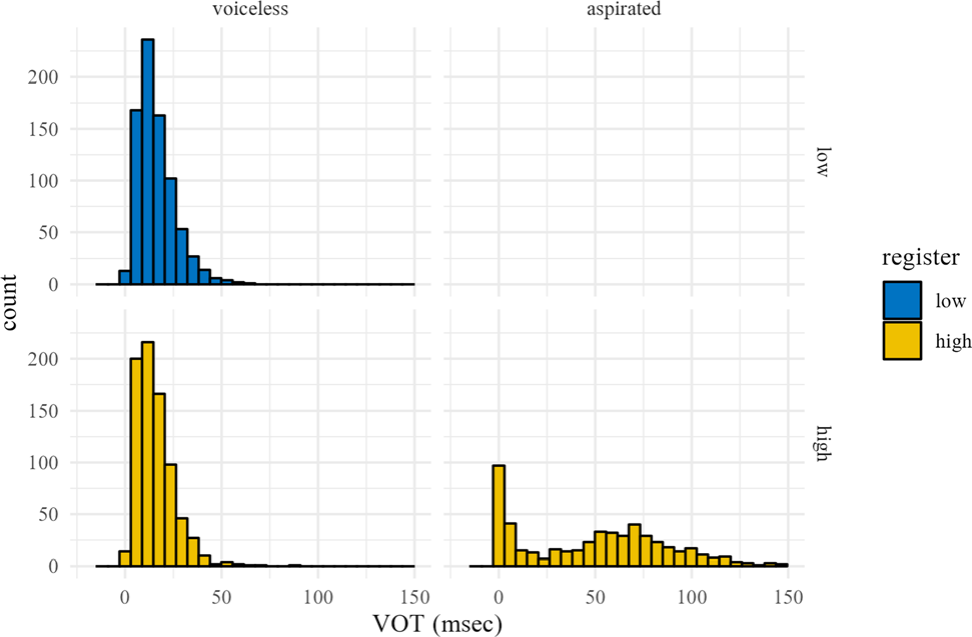
VOT distributions, Northern Kmhmu’.

Table 5 shows descriptive statistics for VOT for Northern Kmhmu’, plotted in Figure 7. While there is clear difference between plain voiceless and aspirated plosives, there is no observable effect of register on VOT within the class of voiceless plosives. We identified only a single instance (out of 666) of true passive voicing of a voiceless plosive.

**Table 5:** VOT and voice lag by voicing and onset articulation (in msec), Northern Kmhmu’.

Register	Onset	Mean VOT	Median VOT	SD VOT
High	t	10	9	6
Low		11	10	5
High	k	20	18	10
Low		21	19	10
High	t^h^	58	60	28
	k^h^	44	45	43

#### 3.1.2 Rhotics

**Figure 8:**
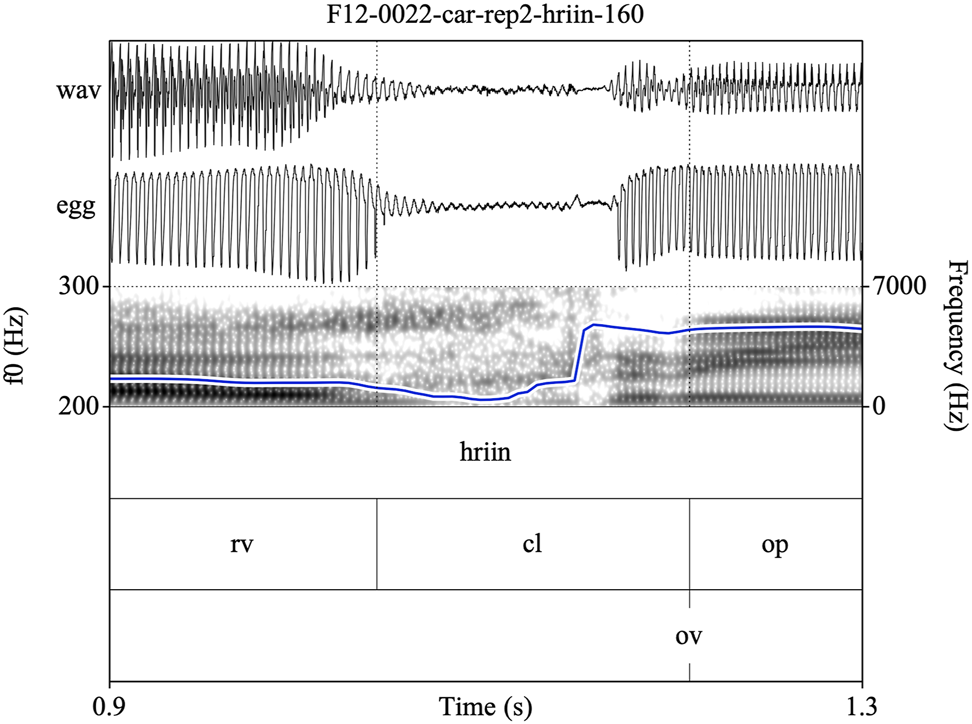
A high-register /rii/ template item /^h^riːn/ ‘to support, lead with hands’, Eastern speaker EF12.

**Figure 9:**
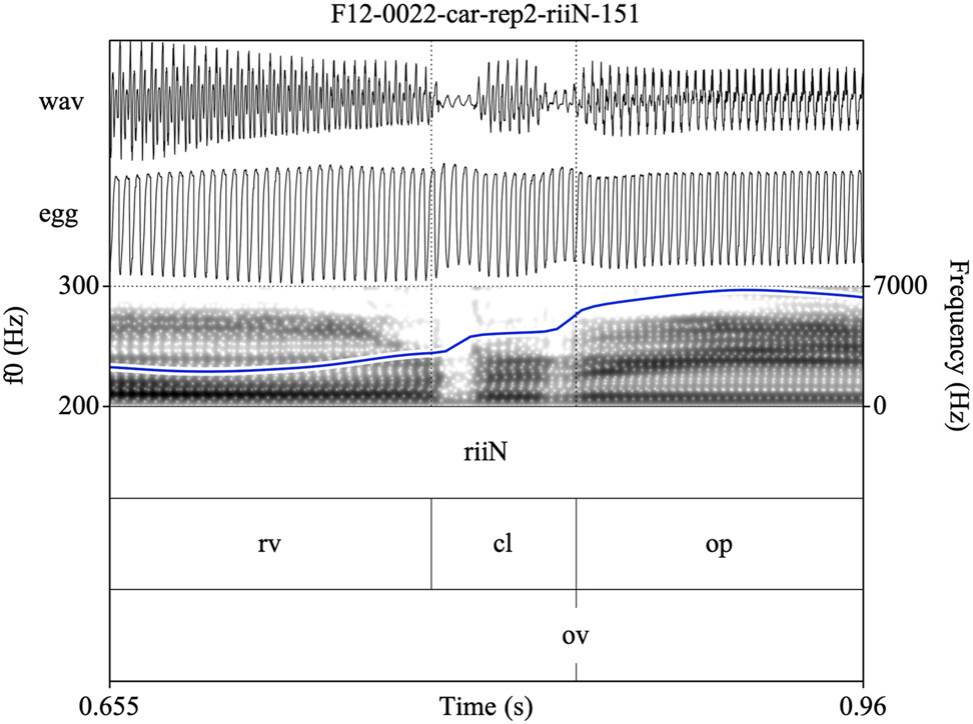
A low-register /rii/-template item /riːŋ/ ‘sing (cricket)’, Eastern speaker EF12.

Eastern Kmhmu’ retains a contrast between voiced and voiceless rhotics (as well as other sonorants), as illustrated by the examples in Figures 8 and 9. Voiceless rhotics in Eastern Kmhmu’ are typically characterized by a period of frication noise, often accompanied by some passive vocal fold vibration, followed by a short trill or rhotic approximant (Figure 8). Voiced rhotics, on the other hand, are characterized by vocal fold vibration throughout the trill or approximant (Figure 9).

**Figure 10:**
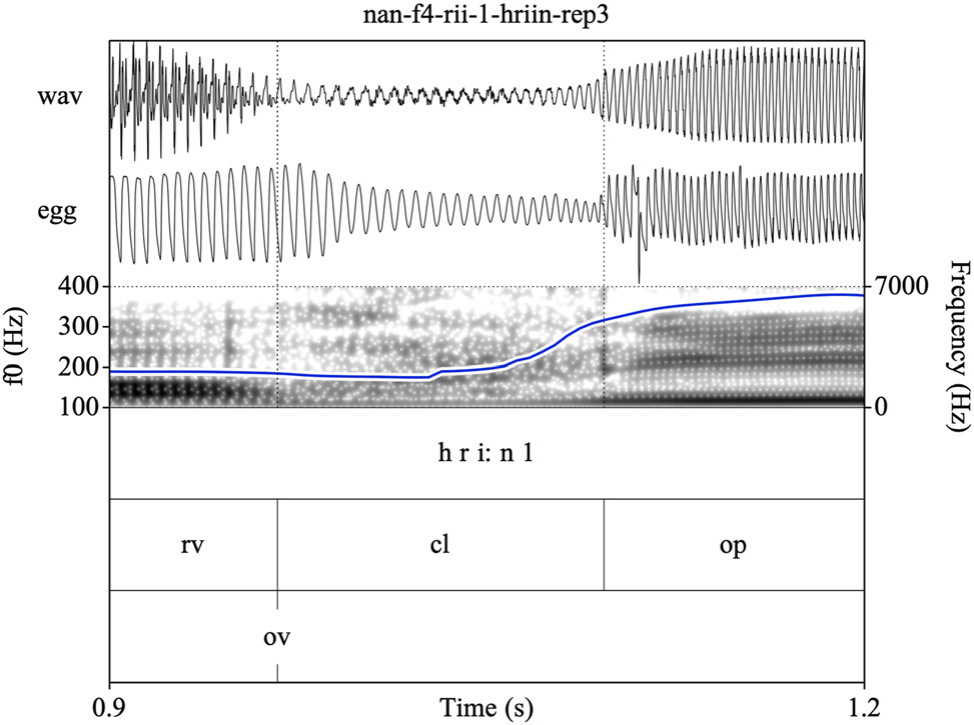
A high-register /rii/ template item, /riːn/ ‘to lead s.o. by the hands’, Northern Kmhmu’ speaker NF4.

**Figure 11:**
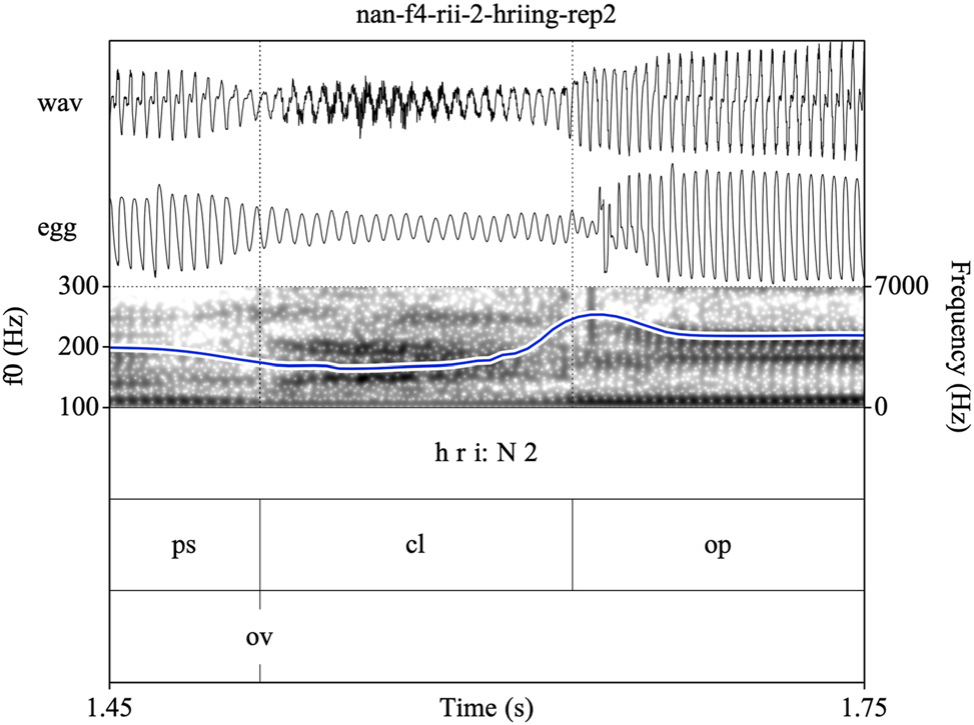
A low-register /rii/ template item /riːŋ/ ‘sing (cricket)’, Northern Kmhmu’ speaker NF4.

As Northern Kmhmu’ is widely reported to have neutralized the voicing contrast in sonorants, we were somewhat surprised to hear what sounded like voiceless sonorants during initial exploratory work with our Northern Kmhmu’ consultants. This led us to transcribe several items, both high- and low-register, as voiceless, as shown in Figures 10 and 11. However, upon closer inspection of the audio files, we found that there is no consistent acoustic basis for these transcriptions; rather, there is simply a spectrum of variable realization of /r/ in this Northern Kmhmu’ dialect, comparable to that seen in languages such as Khmer (Kirby 2014). For some speakers, all rhotics were mostly voiced; for others, they were mostly voiceless. As an attempt to get a handle on the scope of variation, we calculated the proportion of trackable f0 during the closure for all voiced and (in EK) voiceless sonorants, as well as the mean CPP values during the closure. While clearly imperfect, these back-of-the-envelope calculations provide a way to estimate the extent of glottal pulsing during the closure, along with the general degree of breathiness (since glottal pulsing and aspiration noise are not mutually incompatible). For EK, the average proportion of trackable f0 during /r/ was 93% (SD 19) and during /^h^r/ was 81% (SD 26), whereas for NK, f0 was on average trackable over 84% (SD 29) of the closure for tokens we had coded as (low-register) /r/s, 83% (SD 27) for those coded as (high-register) /^h^r/s, and 71% (SD 33) for those coded as (low-register) /^h^r/s. Greater variation was observed between speakers: for some, f0 was typically measurable throughout the closure, whereas for others, this was closer to 50–60%.

CPP differences between voiced and voiceless sonorants averaged about 7 dB for EK, with low-register (voiced) sonorants having higher values. Most importantly, for NK, mean CPP values were almost identical regardless of whether a token was high or low, or coded as /r/ or /^h^r/ (13.57–13.67 dB, SD 1.98–2.08).

These results are consistent with our impressions that rhotics, especially in our NK sample, are generally produced with a greater or lesser degree of turbulence, but do not suggest that NK retains a voicing distinction in the rhotics. Since our primary interest is in identifying potential acoustic correlates of register, we treat all NK rhotics as belonging to the low register in our subsequent analyses.

### 3.2 Vowels

#### 3.2.1 F0

**Figure 12:**
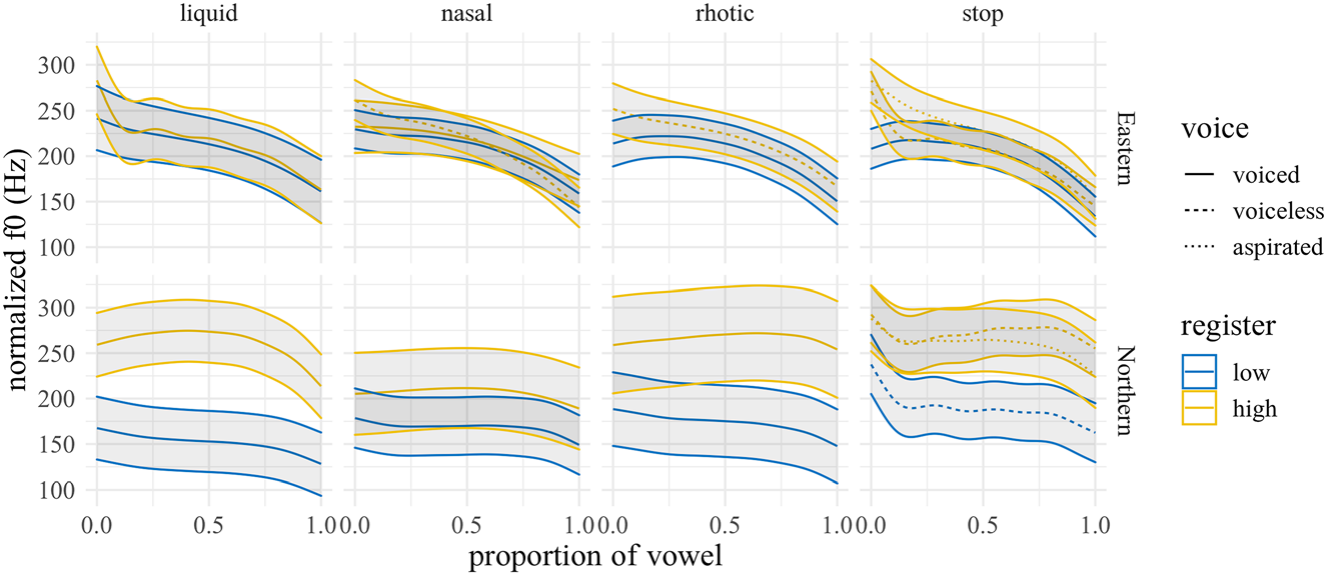
Predicted f0 trajectories (in Hz) over vowels for different levels of voicing and register by manner and dialect. Shading indicates 95% confidence intervals around the means.

**Figure 13:**
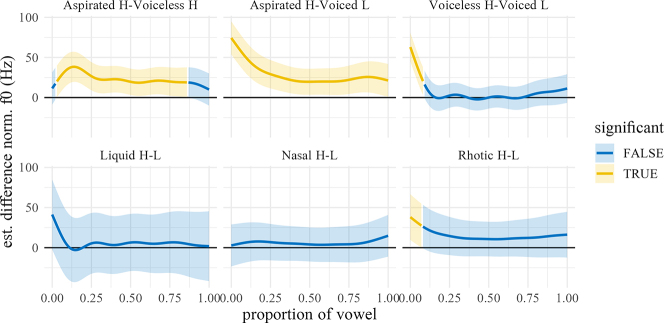
Difference smooths f0 between different pairs of Manner.Reg.Voice levels, Eastern Kmhmu’. Confidence intervals are shaded blue when overlapping with zero; yellow intervals represent statistically significant differences between levels.

**Figure 14:**
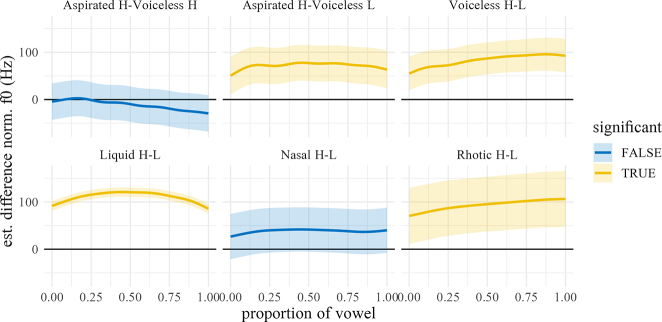
Difference smooths for f0 between different pairs of Manner.Reg.Voice levels, Northern Kmhmu’. Confidence intervals are shaded blue when overlapping with zero; yellow intervals represent statistically significant differences between levels.

The predicted f0 trajectories over the vowel by manner and dialect are shown in Figure 12, with difference smooths in Figure 13 (EK) and Figure 14 (NK). In EK, there is a substantial normalized f0 difference between (high-register) voiceless aspirated and (low-register) voiced plosives at 10% into the vowel (40 Hz, SE = 7.94, *t* = 5.09), reduced to 18 Hz at vowel midpoint (SE = 6.79, *t* = 2.59). Differences between voiceless aspirated and unaspirated plosives, while not as pronounced, were still estimated to be 24 Hz at vowel onset (SE = 8.05, *t* = 2.99) and were similarly reduced by around 50% at midpoint (13 Hz, SE = 6.84, *t* = 1.89). The difference between (high-register) voiceless unaspirated and (low-register) voiced plosives is around 16 Hz at the 10% point (SE = 7.18, *t* = 2.28) but is negligible by midpoint (5 Hz, SE = 5.99, *t* = 0.78). Average differences between sonorants of different registers range from 10 to 26 Hz at 10% of the vowel, but differences by midpoint were not significant for any manner (see Figure 13 and Appendix C).

In NK, the estimated marginal mean differences between registers are considerable for all manners, ranging from 34 Hz (nasals) to 103 Hz (liquids) at onset and from 40 Hz (nasals) to 120 Hz (liquids) at midpoint.5The difference smooths between high and low-register nasals indicate that these differences are not significant. This is due to the fact that the items /cŋaːl/ and /pŋaːr/, which are classified as high register (due to register spreading), are now realized by most speakers as low register. Similarly, among the liquids, /lɔːjt/ is now frequently realized as low-register by at least some speakers in our sample. The difference in f0 between high and low register voiceless plosives actually increases from 67 Hz at onset to 87 Hz at midpoint.

#### 3.2.2 Phonation: H1*–H2*

**Figure 15:**
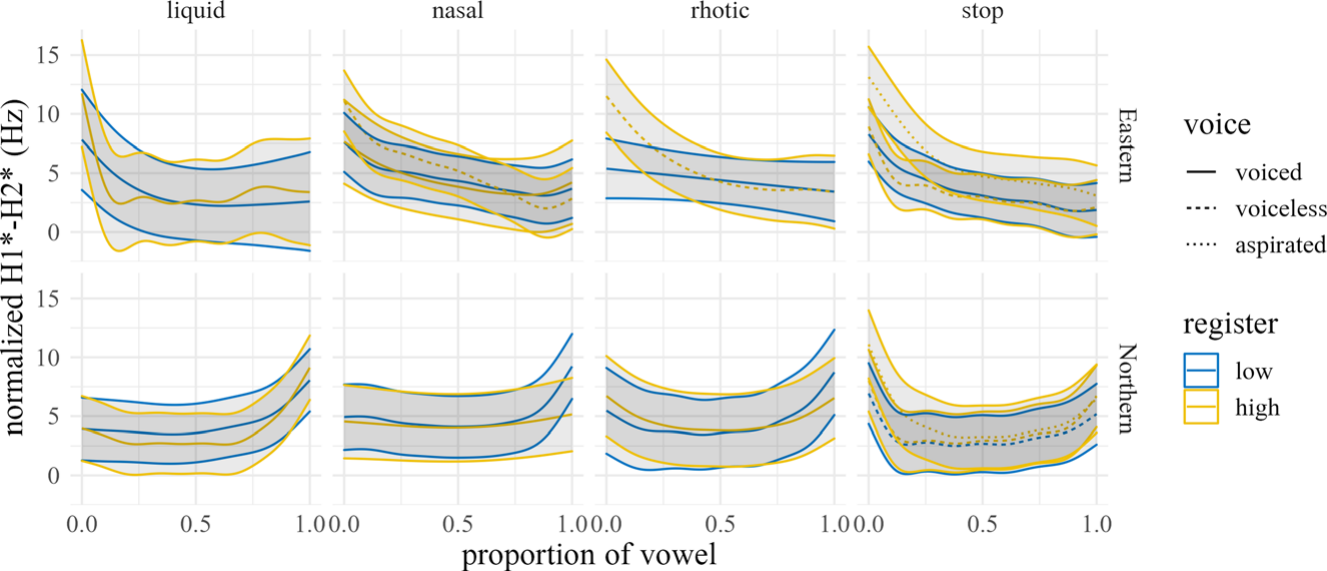
Predicted H1*–H2* trajectories (in dB) over vowels for different levels of voicing and register by manner and dialect. Shading indicates 95% confidence intervals around the means.

**Figure 16:**
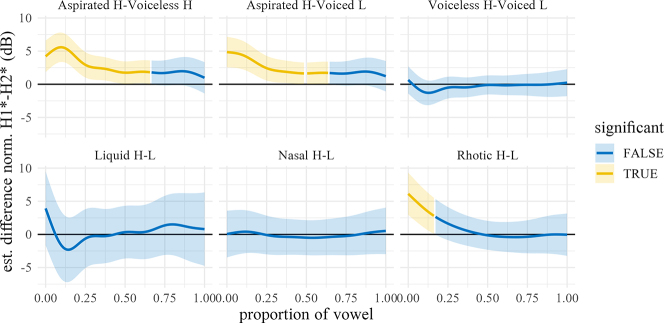
Difference smooths for H1*–H2* between different pairs of Manner.Reg.Voice levels, Eastern Kmhmu’. Confidence intervals are shaded blue when overlapping with zero; yellow intervals represent statistically significant differences between levels.

**Figure 17:**
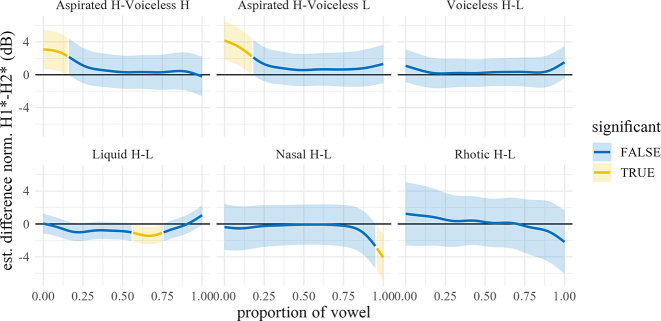
Difference smooths for H1*–H2* between different pairs of Manner.Reg.Voice levels, Northern Kmhmu’. Confidence intervals are shaded blue when overlapping with zero; yellow intervals represent statistically significant differences between levels.

The normalized H1*–H2* trajectories of the vowel (averaged over speakers and repetitions within manner and dialect) are shown in Figure 15, with difference smooths for each variety shown in Figures 16 and 17. In EK, differences between voiced and voiceless unaspirated plosives, and high and low register nasals and liquids, are negligible (1–2.5 dB) and probably inaudible (Garellek et al. 2016, citing Garellek et al. 2013, report a JND for H1–H2 of 4.1 dB; Kreiman et al. 2010 report 3.61 dB for English and 2.6 dB for Gujarati listeners). Aspirated plosives have H1*–H2* of around 5.5 dB greater than unaspirated and voiced plosives at vowel onset, as expected due to the increased glottal opening, but difference at vowel midpoint is only around 2 dB. Similarly, in NK there are only small differences (around 3–4 dB) in this measure between aspirated and unaspirated plosives at vowel onset, again presumably due to the change in glottal width.

#### 3.2.3 Phonation: CPP

**Figure 18:**
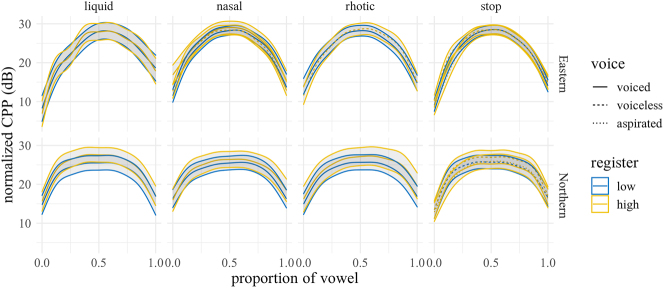
Predicted CPP trajectories (in dB) over vowels for different levels of voicing and register by manner and dialect. Shading indicates 95% confidence intervals around the means.

**Figure 19:**
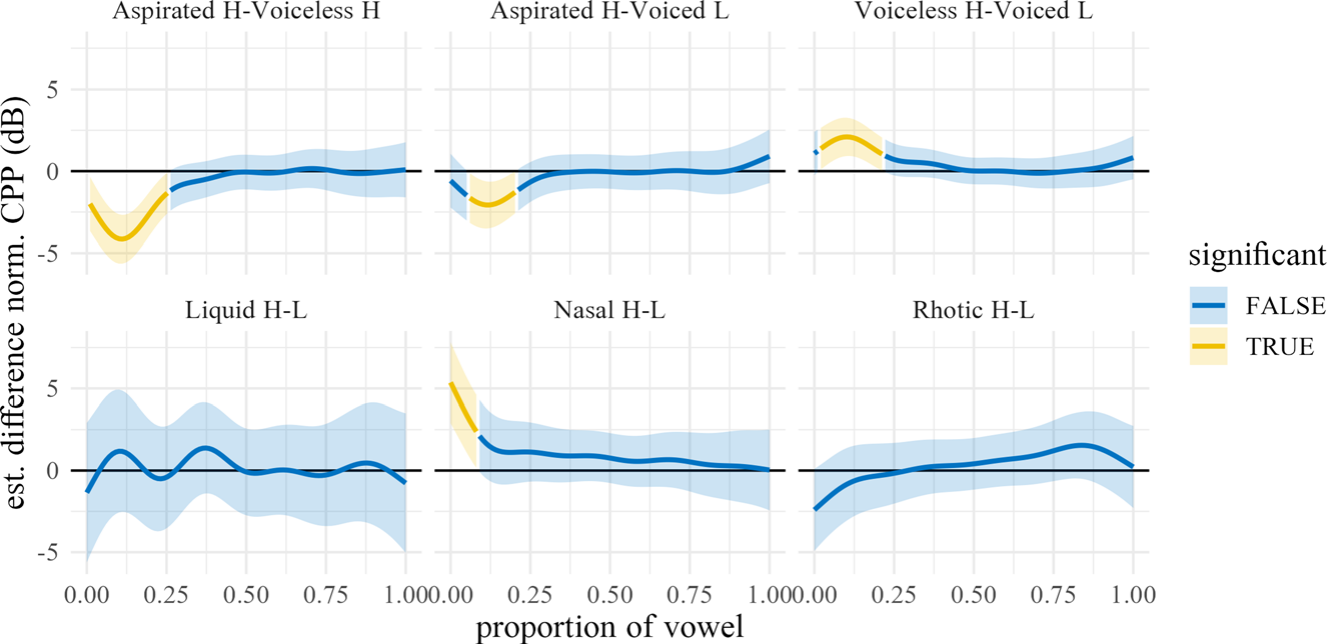
Difference smooths for CPP between different pairs of Manner.Reg.Voice levels, Eastern Kmhmu’. Confidence intervals are shaded blue when overlapping with zero; yellow intervals represent statistically significant differences between levels.

**Figure 20:**
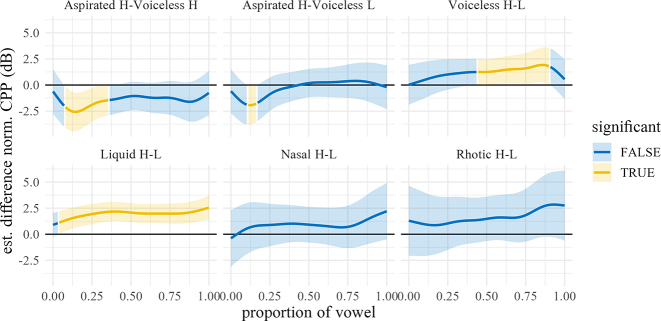
Difference smooths for CPP between different pairs of Manner.Reg.Voice levels, Northern Kmhmu’. Confidence intervals are shaded blue when overlapping with zero; yellow intervals represent statistically significant differences between levels.

Figure 18 shows the predicted trajectories for CPP, with difference smooths following in Figures 19 and 20. In NK, high register sonorants tend to have numerically greater CPP than low register sonorants, especially liquids, although the estimated differences are all <2 dB. This effect may be due to the fact that high-register items were impressionistically often produced with greater vocal intensity, which may correlate with increased CPP (Brockmann-Bauser et al. 2021). In EK, significant effects were only observed at vowel onset between high and low register nasals and plosives (all differences ≤4 dB; see Appendix C).

#### 3.2.4 EGG: closed quotient

**Figure 21:**
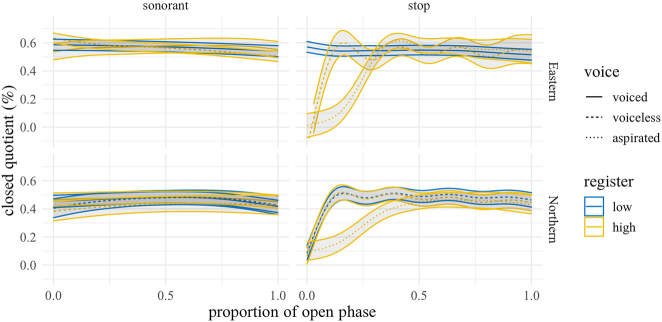
Predicted closed quotient following closure release averaged over speakers, items and repetitions by dialect, manner, voicing, and register. Closing instant determined from dEGG peak; opening instant determined as the time when the negative-going Lx signal crossed an amplitude threshold of 3:7 of that cycle’s peak-to-peak amplitude. See Section 2.4 for details.

**Figure 22:**
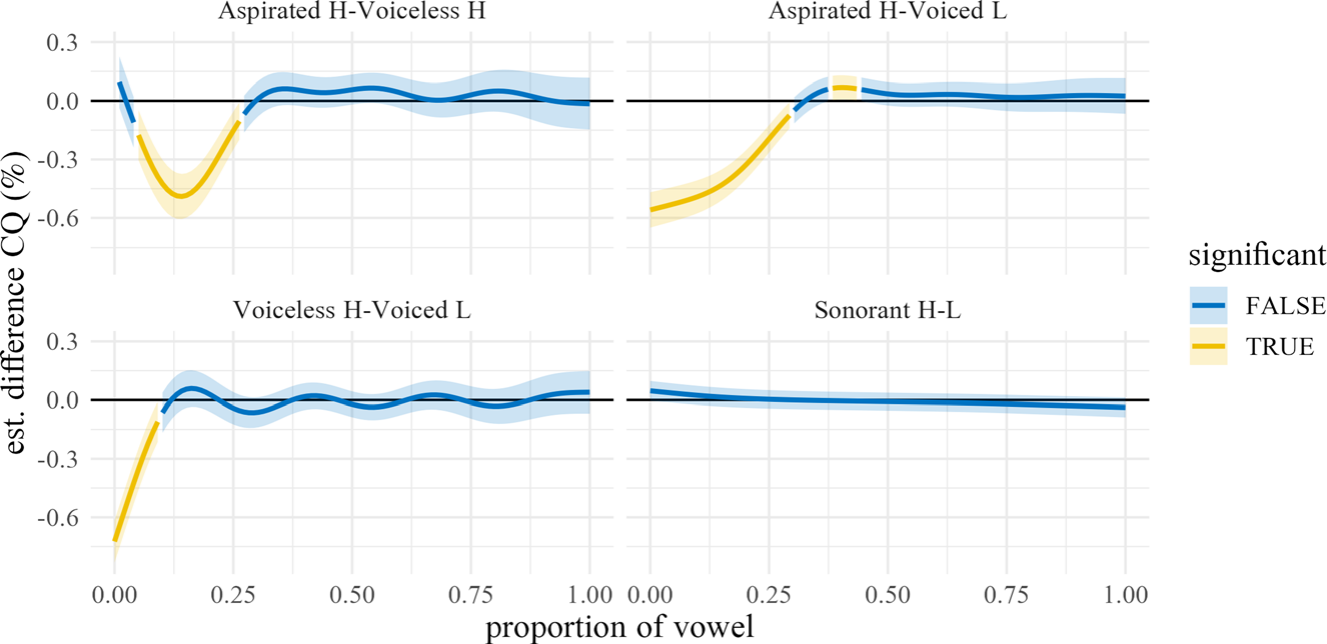
Difference smooths for closed quotient between different pairs of Manner.Reg.Voice levels, Eastern Kmhmu’. Confidence intervals are shaded blue when overlapping with zero; yellow intervals represent statistically significant differences between levels.

**Figure 23:**
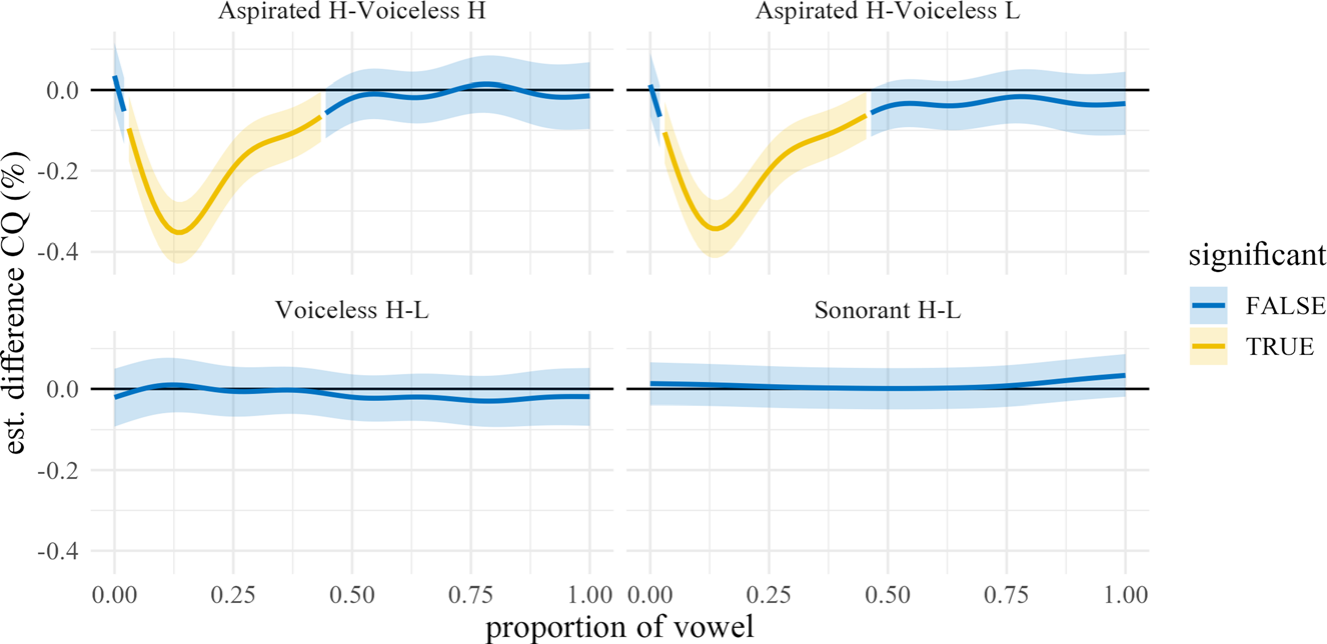
Difference smooths for closed quotient between different pairs of Manner.Reg.Voice levels, Northern Kmhmu’. Confidence intervals are shaded blue when overlapping with zero; yellow intervals represent statistically significant differences between levels.

Figure 21 shows the GAMM-predicted trajectories of the vocal fold closed quotient measured using “Howard’s method”. Here we show time from closure release, rather than voicing onset, as a check on the measure’s general ability to capture the closed quotient (CQ). Since exploratory data analysis indicated no observable differences between rhotics, nasals, and laterals, we merge them and present them as “sonorants”. As expected, CQ is lowest following the release of voiceless aspirated plosives in both varieties; in EK, the mean difference between aspirated and unaspirated at this timepoint is around 42%, while for NK, it is around 31%. However, differences by midpoint are negligible (see Figures 22 and 23 and Appendix C).

#### 3.2.5 Formants

**Figure 24:**
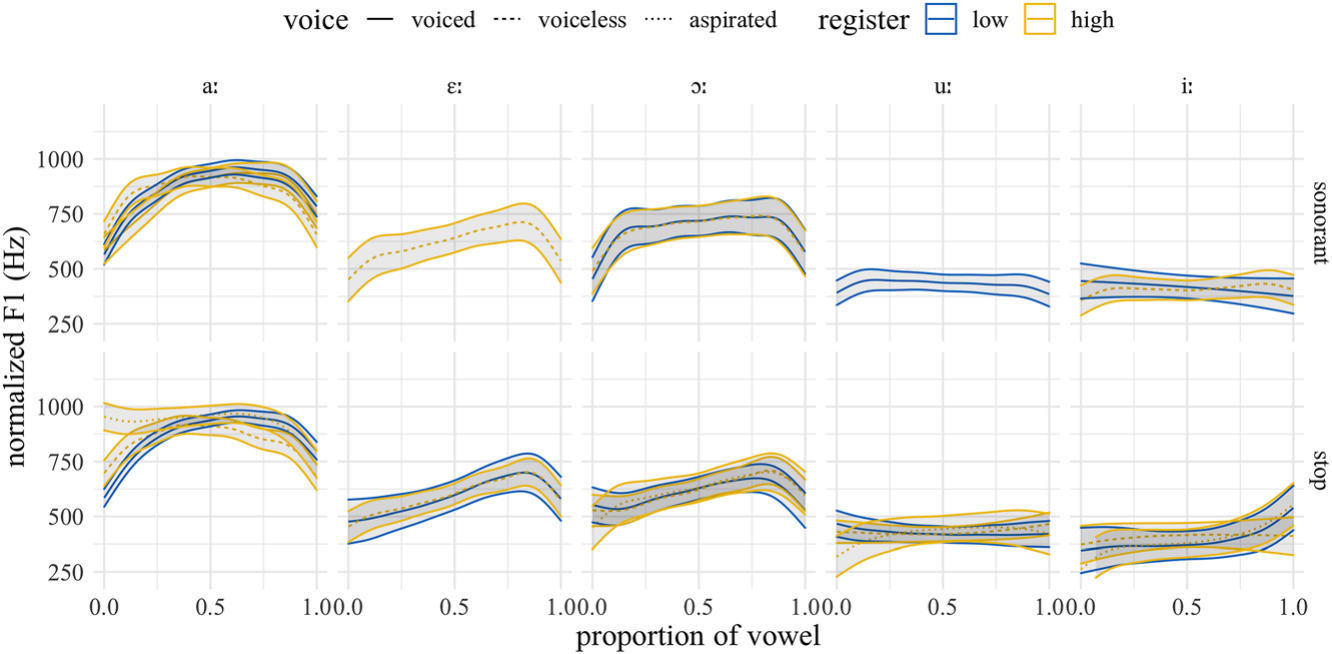
Predicted F1 trajectories (in Hz) over vowels, EK group, averaged over speakers, items and repetitions by manner, voicing, and register.

**Figure 25:**
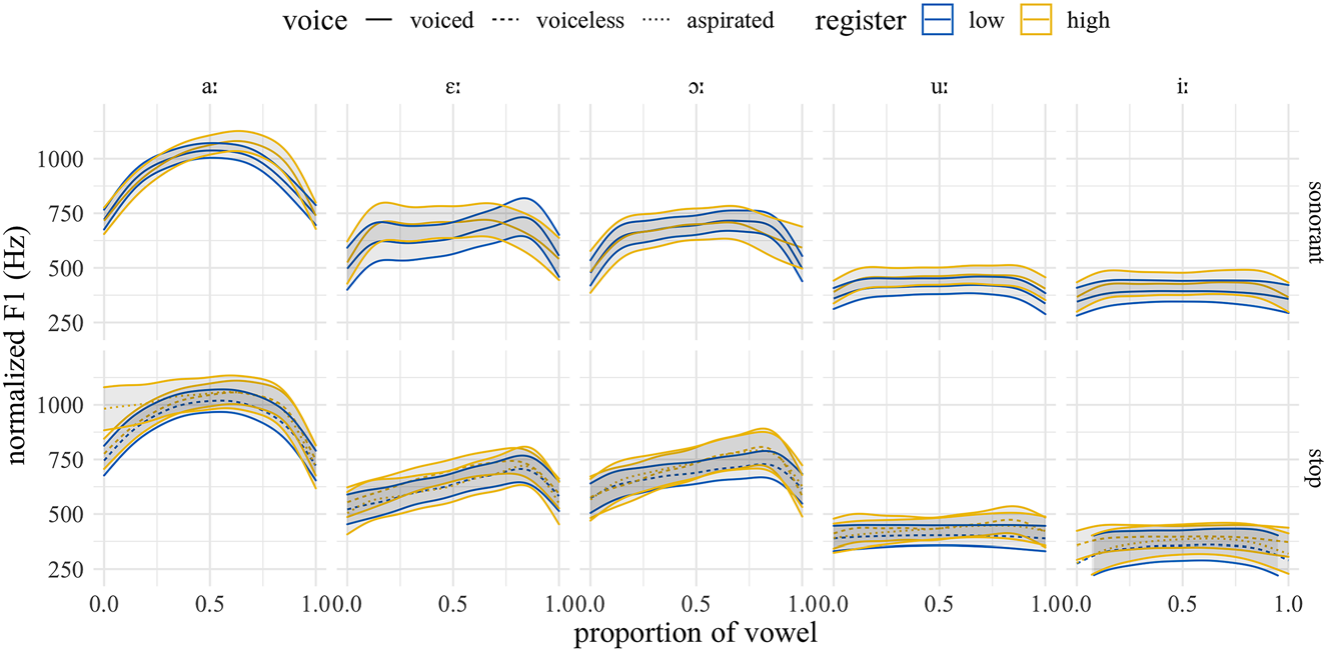
Predicted F1 trajectories (in Hz) over vowels, NK group, averaged over speakers, items and repetitions by manner, voicing, and register.

To facilitate comparison across dialects, we focus here on those vowels for which we have examples in both dialects. As for closed quotient, since exploratory data analysis indicated no observable differences between rhotics, nasals, and laterals, we merge them and present them as “sonorants”. Figure 24 shows the F1 trajectories for EK and Figure 25 for NK. There is some evidence for F1 raising (i.e., vowel lowering) following voiceless aspirated plosives in both varieties, especially for the low vowel /aː/, but this appears to be restricted to aspirated plosives. In NK, there is some indication that vowels following low-register onsets are overall higher (i.e. realized with lower F1), but these differences were not significant in post-hoc pairwise comparisons.

**Figure 26:**
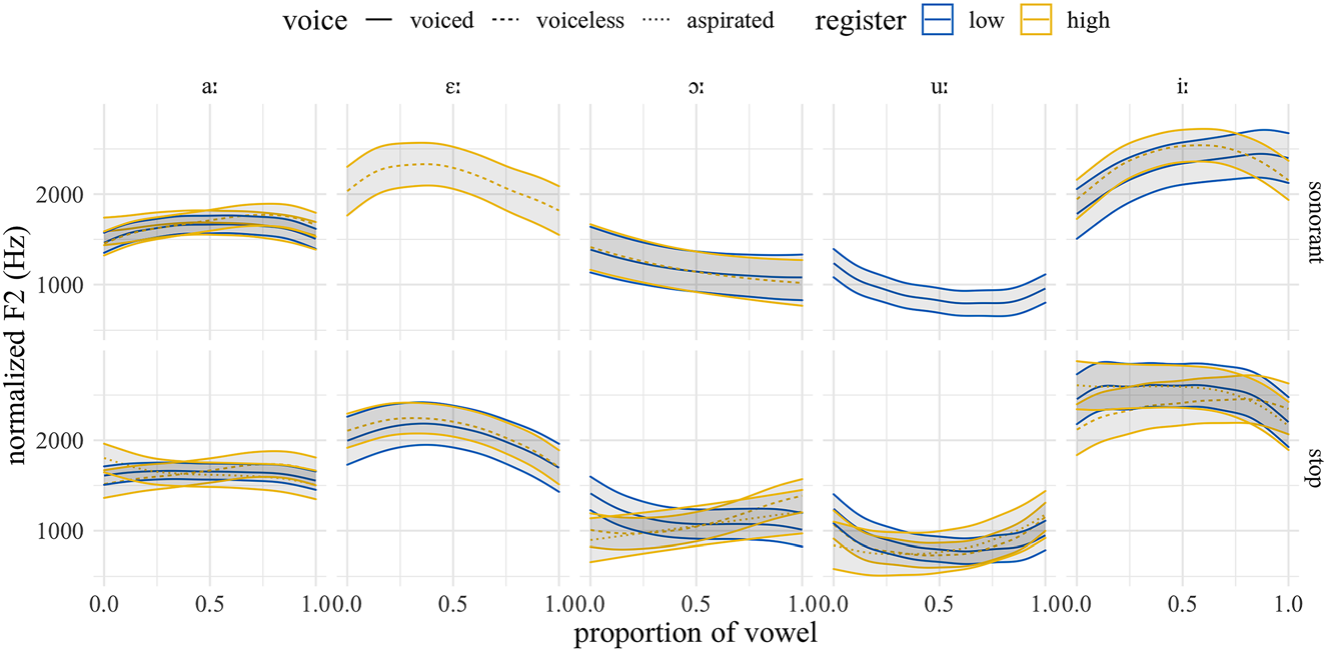
Predicted F2 trajectories (in Hz) over vowels, EK group, averaged over speakers, items and repetitions by manner, voicing, and register.

**Figure 27:**
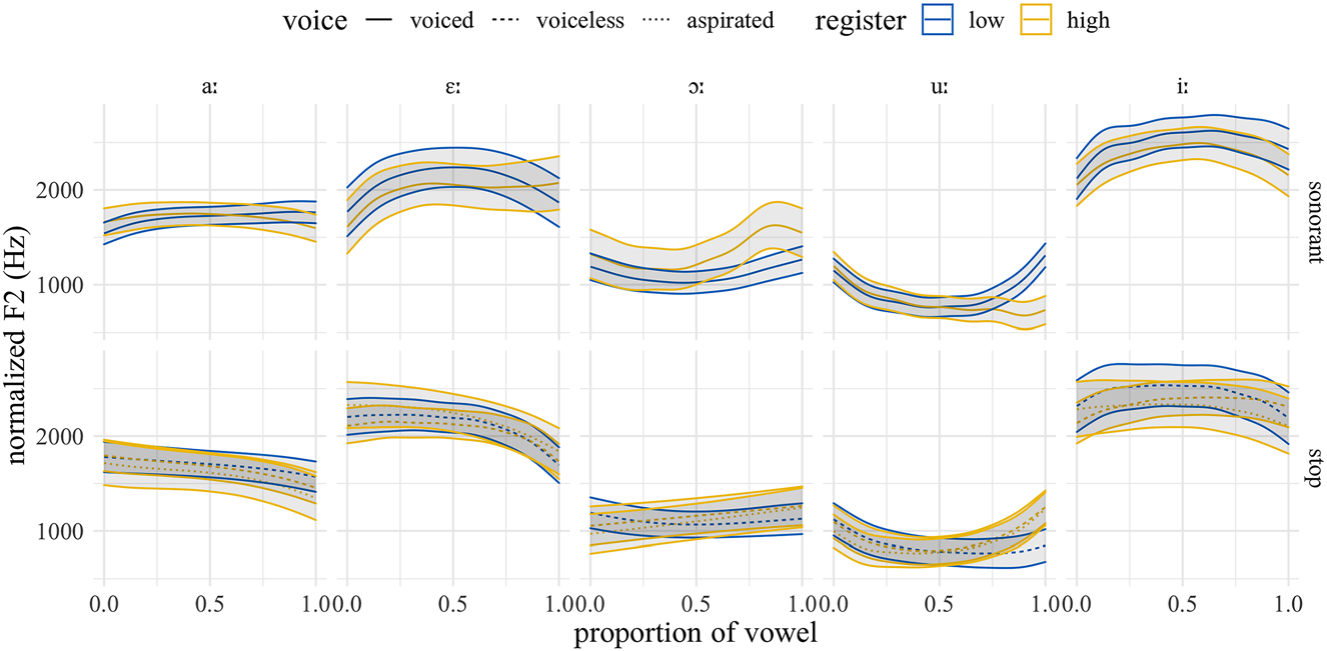
Predicted F2 trajectories (in Hz) over vowels, NK group, averaged over speakers, items and repetitions by manner, voicing, and register.

Figures 26 and 27 show the F2 trajectories for EK and NK respectively. In EK, the differences in f0 for the high vowel /iː/ are likely an artifact of asymmetries in our wordlist, which contains just three items with plosive onsets and the high front vowel. Overall, however, we find no evidence for a robust effect of register on F2 in either variety.

### 3.3 Individual variation and relative role of acoustic cues

As a way to explore possible individual differences in the production of cues, we estimated the magnitude of the difference between (voiced/voiceless unaspirated) plosives of different registers by computing the effect size indicator Cohen’s *d* (Cohen 1988 [1977]) for each speaker and acoustic property. Following Brunelle et al. (2020), we calculated Cohen’s *d* as the vowel-weighted difference between the means of the two registers over the first 10% of the vowel, divided by the pooled register-weighted mean of their standard deviations. While this measure has the advantage of being simple to compute, the results must be interpreted with caution, as possible correlations between cues are not modeled.

**Figure 28:**
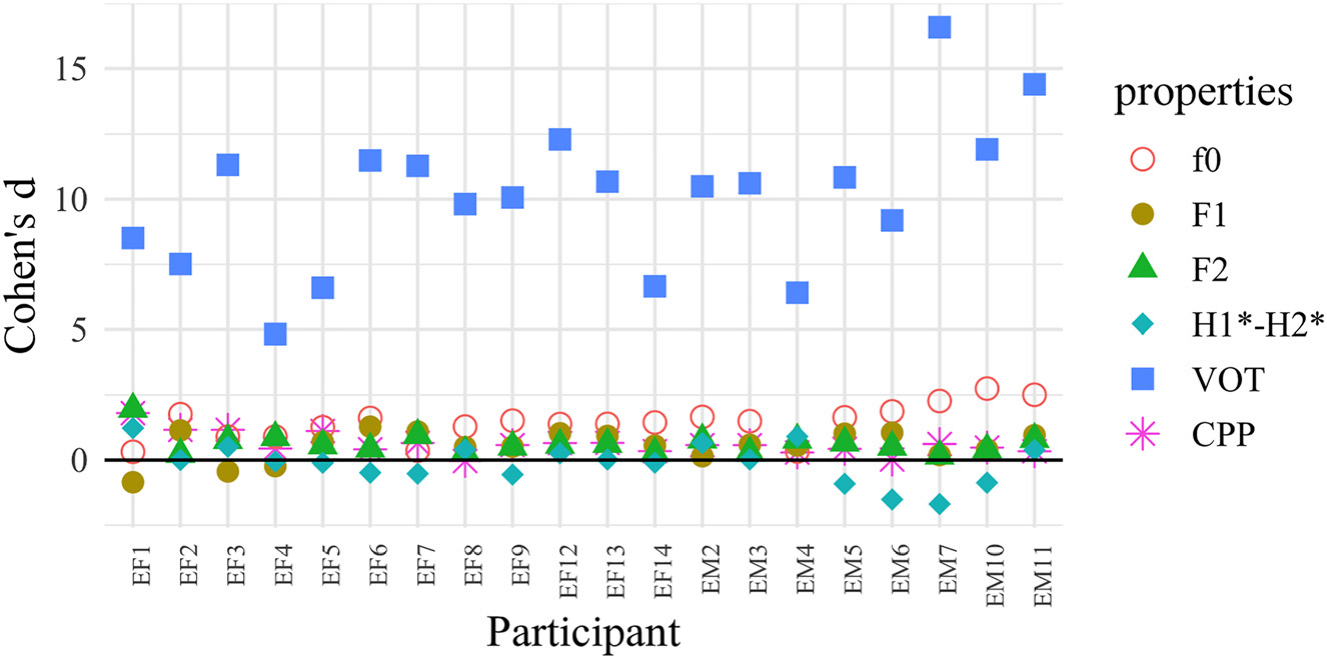
Individual variation in the use of voicing-related acoustic properties distinguishing voiced and voiceless unaspirated stops, EK, expressed as Cohen’s d scores. Cohen’s d for H1*–H2* and F2 have been multiplied by −1. Subject labels encode sex and dialect group.

**Figure 29:**
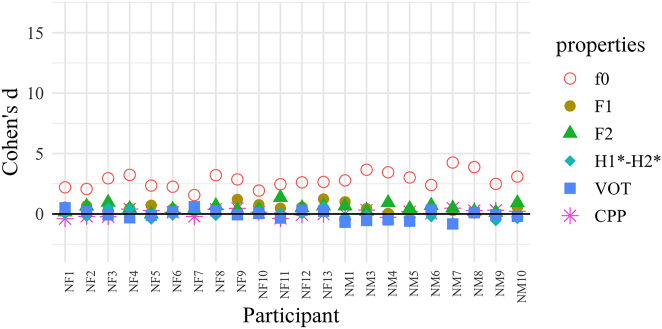
Individual variation in the use of voicing-related acoustic properties distinguishing voiced and voiceless unaspirated stops, NK, expressed as Cohen’s d scores. Cohen’s d for H1*–H2* and F2 have been multiplied by −1. Subject labels encode sex and dialect group.

The Cohen’s *d* scores are plotted separately for each dialect in Figures 28 and 29 for each of the 6 acoustic properties reported in Section 3.1 and 3.2. Scores for H1*–H2* and F2 have been multiplied by −1 so that positive scores represent differences going in the expected direction. Scores below zero thus indicate that the expected correlation between register and the acoustic property in question is reversed.

In EK, VOT is massively more differentiated than all other cues for all speakers (average Cohen’s d of about 10). Yet, f0 is also a non-negligible secondary cue for many speakers. In NK, f0 is clearly the dominant acoustic property. The reason why it is not as categorical as VOT in EK (average Cohen’s d of about 3 vs. 10) may be due to the fact that intonation is known to interact with register-conditioned f0 in Kmhmu’ (Karlsson et al. 2012). Other cues are sometimes close to values of 1 for some speakers, but they are so inconsistent across speakers that they cannot be interpreted with any confidence.

## 4 Discussion and conclusion

### 4.1 Discussion

The analysis of our acoustic and electroglottographic data from Northern and Eastern Kmhmu’ revealed a pattern of results similar to those previously reported for related Kmhmu’ varieties (Abramson et al. 2007; Svantesson and House 2006). In EK, we found the laryngeal contrast signaled by clear differences in VOT, with no evidence of formant or phonation cues. In NK, on the other hand, VOT differences were neutralized, as expected, and the contrast is maintained exclusively by f0 differences on the following vowel. These findings allow us to give clear answers to the research questions posed at the outset:

1) *Is the onset voicing contrast still robust in Eastern Kmhmu’? Are there any remnants of the original voicing contrast in Northern Kmhmu’?*

The contrast between prevoiced and voiceless unaspirated stops in Eastern Kmhmu’ is extremely robust; spontaneous devoicing in our corpus is exceedingly rare. The onset stops of Northern Kmhmu’, on the other hand, preserve no traces of the original voicing contrast in terms of measurable differences in VOT.

2) *Is f0 a robust secondary property in Eastern Kmhmu’ and the primary contrastive property in Northern Kmhmu’?*

Onset f0 is clearly a secondary property of the voicing contrast in Eastern Kmhmu’, which shows the expected raising of f0 following voiceless (aspirated and unaspirated) stops. On the other hand, f0 is unquestionably the primary contrastive property in the NK variety we have studied here.

3) *Does the voicing contrast condition differences in phonation and formants in Eastern Kmhmu’? Is the pitch contrast accompanied by differences in phonation and formants in Northern Kmhmu’?*

Although we did not find any evidence for phonation or formant cues that might support the conservative voicing contrast in EK, the co-intrinsic f0 effect is certainly robust for this speaker sample. As expected, NK seems to mark register with f0 only. It would thus make sense to call it tone, following definitions like that of Hyman (2006: 229; but see Section 4.1.1 below). If it ever looked like EK, it means that all the secondary cues of voicing disappeared and that only f0 was transphonologized. While we cannot say for sure how this transpired, it would be consistent with the view that voicing contrasts can be transphonologized “directly” into f0-based contrasts.

Of course, neither the present results nor the ongoing dearth of acoustic documentation constitutes conclusive evidence that more conventionally “registral” varieties do not, or have never, existed. Premsrirat (2001: 128) notes that at least some speakers may perceive voice quality differences as carrying negative social value within non-Kmhmu’-speaking society, and thus may suppress particular phonetic features of register depending on who they are speaking with. It is also possible that the fact that the NK consultants in both our study as well as that of Abramson et al. (2007) are frequent and fluent users of (Northern) Thai has accelerated the reliance on f0 in their first language. Acoustic documentation of a Kmhmu’ variety in which phonation type is the primary acoustic correlate of a historical voicing contrast (in the sense of Mon or Wa) remains an outstanding goal.

#### 4.1.1 Is Northern Kmhmu’ a tone language?

Svantesson (1983) and Svantesson and House (2006) review a number of arguments against classifying Northern Kmhmu’ dialects that realize the historical voicing contrast purely through f0 differences as “tonal”. The first argument is the existence of register spreading, already mentioned in Section 2.3, whereby the f0 specification of the main syllable vowel is clearly controlled by the register specification of the presyllable: compare /háːn/ ‘to die’ with causative /p-háːn/ ‘cause to die’ and /rə̀h/ ‘rise’ with causative /p-rə́h/ ‘cause to rise’ or /kóh/ ‘to cut’ with nominalized /km̀-nòh/ ‘cut-up salt’ and /kòh/ ‘weed’ with nominalized /km̀-nòh/ ‘weeding period’ (Svantesson and House 2006: 330). Furthermore, unlike in classical Vietnamese or Chinese verse forms, “tone” plays no part in Kmhmu’ poetry (/múːc/ ‘ant’ rhymes with /pùːc/ ‘wine’).

That having been said, evidence that the onset “controls” the f0 specification is probably not in and of itself an argument against typologizing the Northern Kmhmu’ system as tonal. The Tai dialect of Cao Bằng is surely a canonical syllable-tone language by any metric, yet it displays onset-tone co-occurrence restrictions very similar to Northern Kmhmu’ (Pittayaporn and Kirby 2017). We concur with Svantesson and House (2006) that NK is a tone language at the level of phonetic implementation, but whether it should be regarded as tonal in the phonological representation is a terminological and theory-internal issue.

#### 4.1.2 If not phonation, then…?

The fact that there is no evidence for enhancement or retention of voice quality-related secondary acoustic cues in either the conservative or innovative Kmhmu’ varieties is consistent with the hypothesis that f0 can (but need not) transphonologize directly, unmediated through a stage in which phonation type is contrastive. If not mediated through breathy phonation, how might languages like NK arise?

As reviewed in Section 1.2, many of the phonetic properties associated with register systems correspond to the acoustic outcomes of articulatory strategies broadly aimed at circumventing the “Aerodynamic Voicing Constraint” (Ohala 1983, 2011), which requires that an adequate transglottal pressure drop obtain in order to sustain vocal fold vibration. Obstruents, by their very nature, present a challenge in this regard. Overcoming the AVC involves either reducing the closure duration and/or enlarging the supralaryngeal cavity. There are a number of ways this second goal could be achieved; two of the most relevant for the present discussion are pharyngeal expansion by means of tongue root advancement and larynx lowering. The acoustic side-effects of these strategies include many of the canonical properties of register, such as lower F1, lower f0, and steeper spectral slope. Attempts to overcome the AVC could thus produce multiple acoustic correlates of register simultaneously, but which of these a given language (or listener) “selects” could vary.

### 4.2 Conclusion

For a better understanding of the role that phonation plays in tonogenesis, our research looked at acoustic and electroglottographic data on the production of register in (non-tonal) Eastern Kmhmu’ and (tonal) Northern Kmhmu’, two endpoints of the Kmhmu’ dialect continuum. To critically assess the prevalent idea that at an initial stage of tonogenesis voiced onset consonants condition phonetic differences in phonation on the following vowel, we tested if there was evidence for incipient or redundant vowel quality or phonation in their voicing and tone contrasts. We found no significant differences in phonation type measures or vowel formants between register, either in the conservative EK or the tonal NK dialect. These findings are consistent with a model on which f0 can transphonologize directly, without necessarily going through a stage in which phonation type is contrastive.

## Supplementary Material

Supplementary Material

Supplementary Material

Supplementary Material
