# Supplementary material for: Transphonologization of onset voicing: revisiting Northern and Eastern Kmhmu’
Source: Phonetica. 2023 Jan 31;79(6):591–629. doi: 10.1515/phon-2022-0029 (PMC10065200; doi:10.1515/phon-2022-0029)
Supplement: Supplementary file 1 — Supplementary Material [file j_phon-2022-0029_suppl_001.pdf]

Appendix A: Eastern (Vientiane) word list

Item	Template	Register	Lao prompt	Gloss
baː	bVV	low	ເຈົ້າ (ຜູ້ຍິງ)	you (female)
baːr	bVV	low	ສອງ	two (numeral)
buː	bVV	low	ໂພງ, ພອງ. (ແກ້ມ) ປຸ່ງ	puffy; swollen
daː	dVV	low	ທາ	to apply, paint
daːl	dVV	low	ບໍ່ຄົມ	dull
daːŋ	dVV	low	ໂຕຈິຈ້ຽມ/ໂຕຈິກຽມ	lizard
dɛːr	dVV	low	ຄວ່າງ, ຫວ່ານ (ແຫ)	spread out (fishing net)
dɔːm	dVV	low	ອອນຊອນ	beautiful, sweet, natural (sound)
(hn)duːm	dVV	low	ສຸກ	ripe
gaː	gVV	low	ປີນ	climb
gaːŋ	gVV	low	ເຮືອນ	house
giː	gVV	low	ນີ້	here, this
gɔːŋ	gVV	low	ແກງ	soup
guːm	gVV	low	ຝັດເຂົ້າ	to winnow the paddy
kaːl	kVV	high	ກ່ອນ	before
kɛːn	kVV	high	ມັດຕິດແອວ	tighten at the waist
kɔːl	kVV	high	ບັກ	to cut down
kɔːn	kVV	high	ລູກ	offspring, child
kʰaːl	kʰVV	high	ເສັ້ນຕອກ	thin bamboo strip used for making baskets
kʰiː	kʰVV	high	ທີ່ນີ້	here (locative adv.)
kʰɔːl	kʰVV	high	ຜິວປາກ	whistle
kʰuːl	kʰVV	high	ຂົນ	body hair
glaːŋ	lVV	low	ຫີນ	stone
klaːŋ	lVV	high	ນົກອິນຊີ	eagle
maːm	mVV	low	ເລືອດ	blood
məːj	mVV	low	ນ້ຳມັນ	grease, oil
moːj	mVV	low	ໜຶ່ງ	one (numeral)
muːm	mVV	low	ອາບນ້	to take a bath
m̥aːn	mVV	high	ຝັງ	to bury
m̥aːr	mVV	high	ເກືອ	salt
naː	nVV	low	ລາວ (ແມ່ຍິງ)	she (pronoun); rice paddy
(hr)nɔːm	nVV	low	ຕອກ	thin bamboo strip used for tying things
nuːm	nVV	low	ຍ່ຽວ	urine
n̥aːj	nVV	high	ນັ້ນ	that (demonstrative)
n̥ɛːn	nVV	high	ແຂງ, ແໜ້ນ	hard, tight
n̥ɔːŋ	nVV	high	ຍັງ	still, yet, remain

n̥iː	nVV	high	ໜີ້	debt
ŋaːm	ŋVV	low	ຖາງທາງ	to clear a path
cŋaːr	ŋVV	high	ສີເຫຼືອງ	yellow
pŋaːl	ŋVV	high	ອຸ່ນອາຫານ	to warm up slowly
paːl	pVV	high	ປານ	birthmark
pɛːl	pVV	high	ຈັບເບິ່ງ	catch and watch
pəːj	pVV	high	ວີ	fan, blow
puː	pVV	high	ລີບ (ແກ່ນລີບ)	empty rice husk
puːl	pVV	high	ສີ່	four
p^{h}aːn	p^{h}VV	high	ຂ້າ	to kill
raː	rVV	low	ລ້າງ	wash
raːŋ	rVV	low	ດອກໄມ້	flower
riːŋ	rVV	low	ຮ້ອງ (ຈິ່ລໍ)	sing (cricket)
ruː	rVV	low	ດຶງ	pull, drag
r̥aːŋ	rVV	high	ແຂ້ວ	tooth
r̥iːn	rVV	high	ປະຄອງ	support with hands, lead (old people)
r̥oːj	rVV	high	ຜີ	spirit
siːm	sVV	high	ນົກ	bird
taːj	tVV	high	ອ້າຍ	older sibling
tiːŋ	tVV	high	ລົ້ມ (ໄມ້)	to fall down (house, tree)
tuː	tVV	high	ໃສ່ຮ້າຍປ້າຍສີ	to falsely accuse
t^{h}aːk	t^{h}VV	high	ປອກເປືອກຕົ້ນໄມ້	to strip bark off of tree
