# Supplementary material for: Transphonologization of onset voicing: revisiting Northern and Eastern Kmhmu’
Source: Phonetica. 2023 Jan 31;79(6):591–629. doi: 10.1515/phon-2022-0029 (PMC10065200; doi:10.1515/phon-2022-0029)
Supplement: Supplementary file 2 — Supplementary Material [file j_phon-2022-0029_suppl_002.pdf]

Appendix B: Northern (Huay Lao) word list

Item	Template	Register	Thai prompt	Gloss
ɗaːŋ	ɗVV	high	ปาก	clf of fishnet
ɗɔːm	ɗVV	high	ดู	to look at
kaː	kVV	low	ขึ้น (ต้นไม้)	to climb
kaːl	kVV	high	ปิ้ง, ย่าง	grill something above the fire
kɛːm	kVV	low	ข้างๆ	beside, side
kɛːn	kVV	high	เหน็บเอว (ผ้า)	tighten (at the waist)
kiː	kVV	high	ที่นี่	here
siŋkiː	kVV	low	เมื่อวานนี้	yesterday
kɔːn	kVV	high	ลูก	offspring
kɔːŋ	kVV	low	ภูเขา	ridge, mountain range
kuːl	kʰVV	high	พายุ	storm
kuːm	kʰVV	low	ฝัดข้าวสาร	to winnow in a thin plaited flat basket
kʰaːm	kʰVV	high	ข้าม	cross (water or pond)
kʰɛːn	kʰVV	high	แคน	reed
kʰɔːl	kʰVV	high	ผิวปาก	whistle
kʰuːl	kʰVV	high	ขน	body hair
klaːŋ	lVV	low	หิน, กรวด	stone, pebbles, marble, rock
lɛː	lVV	high	ครึ้มๆ (ท้องฟ้า)	dark (sky before rain)
lɛːn	lVV	low	แลน	chameleon
lɔːjt	lVV	high	หล่อ (ไม้)	let logs fall down on a slope
lɔː	lVV	low	ล้อ	wheel
luː	lVV	high	ลาบเลือด	kind of food (laap)
luː	lVV	low	หอน	howl (dog)
naː	nVV	low	นา	wet-rice field
niː	nVV	high	หนี้	debt
nɔːm	nVV	low	ตอก	thin bamboo string for tying things
nuːm	nVV	low	ปัสสาวะ	urine
ŋaː	ŋVV	low	งา	tusk
cŋaːl	ŋVV	high	เหลือง	yellow
pŋaːl	ŋVV	high	อุ่น (แกง, ข้าว)	to warm up (food)

plɛŋiː	ŋVV	low	มะม่วง	mango
ŋɔː	ŋVV	low	ง้อ	to make up after a fight
ŋuːm	ŋVV	low	นกงูม	kind of bird
raː	rVV	low	ล้าง	to wash
raːŋ	rVV	high	ฟัน	tooth
raːŋ	rVV	low	ดอกไม้	flower
riːn	rVV	high	จูงมือ	lead s.o. by hands
riːŋ	rVV	low	ร้อง(จิ้งหรีด)	sing (cricket)
ruː	rVV	low	ดึง, ลาก	pull, drag
saː	sVV	high	ตะกร้า	basket
sɛː	sVV	high	เจาะ (รูเล็กของแข็งไม่มาก)	punch a small hole
siːm	sVV	high	นก	bird
suː	sVV	high	สู่ขวัญ	blessing ceremony
taːm	tVV	high	ตีมีด	to hit (knife, rice)
taːl	tVV	low	ทื่อ (มีด)	dull (blade)
tɛːm miaŋ	tVV	high	ปั้น (เมี่ยง, หมาก)	wrap k.o. food
tɛːl	tVV	low	หว่าน (แห)	spread out (fishing net)
tiːŋ	tVV	high	ล้ม (ต้นไม้)	fall down (tree)
tɔːl	tVV	low	กระบอกรองตีเหล็ก	anvil
tuːn	tVV	high	ตุ่น	mole
tuːm	tVV	low	สุก (ผลไม้)	to be ripe
t^hiː	t^hVV	high	ตระหนี่	stingy
t^huː	t^hVV	high	ถู (ฟัน)	brush (teeth)
