# Supplementary material for: Transphonologization of onset voicing: revisiting Northern and Eastern Kmhmu’
Source: Phonetica. 2023 Jan 31;79(6):591–629. doi: 10.1515/phon-2022-0029 (PMC10065200; doi:10.1515/phon-2022-0029)
Supplement: Supplementary file 3 — Supplementary Material [file j_phon-2022-0029_suppl_003.pdf]

Appendix C: GAMM estimates and estimated marginal means

Table 1: VOT and voicing lag times, Eastern Kmhmu'.

register	onset	mean.VOT	sd.VOT	mean.lag	sd.lag
low	b	-79.32	28.72	7.02	7.62
low	d	-86.51	41.40	8.81	5.72
low	g	-80.49	28.62	15.85	11.12
high	k	17.87	11.37		
high	kʰ	70.94	22.01		
high	p	9.02	5.43		
high	pʰ	49.00	32.05	11.01	
high	t	9.82	6.26		
high	tʰ	59.31	23.02		

Table 2: VOT and voicing lag times, Northern Kmhmu'.

register	onset	mean.VOT	sd.VOT	mean.lag	sd.lag
low	b	-79.32	28.72	7.02	7.62
low	d	-86.51	41.40	8.81	5.72
low	g	-80.49	28.62	15.85	11.12
high	k	17.87	11.37		
high	kʰ	70.94	22.01		
high	p	9.02	5.43		
high	pʰ	49.00	32.05	11.01	
high	t	9.82	6.26		
high	tʰ	59.31	23.02		

Table 3: Parametric coefficient estimates for f0 GAMM model, Eastern Kmhmu'.

	Estimate	Std. Error	t value	Pr(>|t|)
(Intercept)	210.53	8.66	24.32	0.00
manner.reg.voice1	-0.63	6.21	-0.10	0.92
manner.reg.voice2	-1.35	3.05	-0.44	0.66
manner.reg.voice3	-2.50	1.58	-1.58	0.11
manner.reg.voice4	2.04	2.63	0.78	0.44
manner.reg.voice5	1.19	1.62	0.73	0.46
manner.reg.voice6	0.97	0.86	1.12	0.26
manner.reg.voice7	1.34	1.03	1.30	0.19
manner.reg.voice8	-0.80	0.61	-1.32	0.19
manner.reg.voice9	1.62	0.66	2.44	0.01

Table 4: Estimated marginal mean differences in f0 over first 10% of vowel between manner/register/voicing pairs, Eastern Kmhmu'.

contrast	estimate	SE	t.ratio	p.value
liquid.low.voiced - liquid.high.voiced	-2.22	20.99	-0.11	1.00
nasal.low.voiced - nasal.high.voiced	-6.66	12.59	-0.53	1.00
nasal.low.voiced - nasal.high.voiceless	-23.86	8.88	-2.69	0.18
rhotic.low.voiced - rhotic.high.voiceless	-23.91	13.67	-1.75	0.77
stop.low.voiced - stop.high.voiceless	-12.87	8.71	-1.48	0.90
stop.low.voiced - stop.high.aspirated	-48.60	10.05	-4.84	0.00
nasal.high.voiced - nasal.high.voiceless	-17.20	12.62	-1.36	0.94

contrast	estimate	SE	t.ratio	p.value
stop.high.voiceless - stop.high.aspirated	-35.73	9.82	-3.64	0.01

Table 5: Estimated marginal mean differences in f0 at 50% of vowel between manner/register/voicing pairs, Eastern Kmhmu'.

contrast	estimate	SE	t.ratio	p.value
liquid.low.voiced - liquid.high.voiced	-6.46	18.20	-0.35	1.00
nasal.low.voiced - nasal.high.voiced	-4.06	10.84	-0.37	1.00
nasal.low.voiced - nasal.high.voiceless	-6.84	7.61	-0.90	1.00
rhotic.low.voiced - rhotic.high.voiceless	-10.74	11.01	-0.98	0.99
stop.low.voiced - stop.high.voiceless	-0.03	7.04	-0.00	1.00
stop.low.voiced - stop.high.aspirated	-19.66	8.28	-2.38	0.34
nasal.high.voiced - nasal.high.voiceless	-2.79	10.79	-0.26	1.00
stop.high.voiceless - stop.high.aspirated	-19.63	8.36	-2.35	0.36

Table 6: Parametric coefficient estimates for f0 GAMM model, Northern Kmhmu'.

	Estimate	Std. Error	t value	Pr(>|t|)
(Intercept)	216.56	11.22	19.30	0.00
manner.reg.voice1	8.41	9.60	0.88	0.38
manner.reg.voice2	4.46	6.68	0.67	0.50
manner.reg.voice3	24.58	3.13	7.84	0.00
manner.reg.voice4	3.55	4.39	0.81	0.42
manner.reg.voice5	12.38	4.27	2.90	0.00

	Estimate	Std. Error	t value	Pr(>|t|)
manner.reg.voice6	-2.37	2.07	-1.15	0.25
manner.reg.voice7	8.62	1.71	5.03	0.00
manner.reg.voice8	5.47	1.77	3.08	0.00

Table 7: Estimated marginal mean differences in f0 over first 10% of vowel between manner/register/voicing pairs, Northern Kmhmu'.

contrast	estimate	SE	t.ratio	p.value
liquid.low.voiced - liquid.high.voiced	-103.27	5.11	-20.20	0.00
nasal.low.voiced - nasal.high.voiced	-33.67	24.31	-1.39	0.90
rhotic.low.voiced - rhotic.high.voiced	-77.61	30.13	-2.58	0.20
stop.low.voiceless - stop.high.voiceless	-67.08	17.89	-3.75	0.01
stop.low.voiceless - stop.high.aspirated	-68.10	20.04	-3.40	0.02
stop.high.voiceless - stop.high.aspirated	-1.02	19.55	-0.05	1.00

Table 8: Estimated marginal mean differences in f0 at 50% of vowel between manner/register/voicing pairs, Northern Kmhmu'.

contrast	estimate	SE	t.ratio	p.value
liquid.low.voiced - liquid.high.voiced	-120.70	4.97	-24.27	0.00
nasal.low.voiced - nasal.high.voiced	-41.56	23.92	-1.74	0.72
rhotic.low.voiced - rhotic.high.voiced	-95.35	29.62	-3.22	0.03
stop.low.voiceless - stop.high.voiceless	-86.94	17.21	-5.05	0.00

contrast	estimate	SE	t.ratio	p.value
stop.low.voiceless - stop.high.aspirated	-77.21	19.29	-4.00	0.00
stop.high.voiceless - stop.high.aspirated	9.73	19.02	0.51	1.00

Table 9: Parametric coefficient estimates for H1-H2 GAMM model, Eastern Kmhmu'.

	Estimate	Std. Error	t value	Pr(>|t|)
(Intercept)	4.31	0.84	5.10	0.00
manner.reg.voice1	0.62	0.67	0.92	0.36
manner.reg.voice2	0.19	0.30	0.65	0.52
manner.reg.voice3	-0.12	0.16	-0.75	0.45
manner.reg.voice4	-0.09	0.27	-0.34	0.74
manner.reg.voice5	0.09	0.19	0.49	0.62
manner.reg.voice6	0.18	0.11	1.65	0.10
manner.reg.voice7	0.16	0.11	1.39	0.16
manner.reg.voice8	-0.11	0.06	-1.84	0.07
manner.reg.voice9	0.17	0.06	2.69	0.01

Table 10: Estimated marginal mean differences in H1-H2 over first 10% of vowel between manner/register/voicing pairs, Eastern Kmhmu'.

contrast	estimate	SE	t.ratio	p.value
liquid.low.voiced - liquid.high.voiced	1.68	2.59	0.65	1.00
nasal.low.voiced - nasal.high.voiced	-0.40	1.67	-0.24	1.00
nasal.low.voiced - nasal.high.voiceless	-2.27	1.26	-1.80	0.73

contrast	estimate	SE	t.ratio	p.value
rhotic.low.voiced - rhotic.high.voiceless	-3.91	1.48	-2.64	0.20
stop.low.voiced - stop.high.voiceless	1.19	0.97	1.22	0.97
stop.low.voiced - stop.high.aspirated	-4.40	1.10	-4.01	0.00
nasal.high.voiced - nasal.high.voiceless	-1.87	1.69	-1.11	0.98
stop.high.voiceless - stop.high.aspirated	-5.58	1.13	-4.94	0.00

Table 11: Estimated marginal mean differences in H1-H2 at 50% of vowel between manner/register/voicing pairs, Eastern Kmhmu'.

contrast	estimate	SE	t.ratio	p.value
liquid.low.voiced - liquid.high.voiced	-0.34	2.03	-0.17	1.00
nasal.low.voiced - nasal.high.voiced	0.49	1.35	0.37	1.00
nasal.low.voiced - nasal.high.voiceless	-0.85	1.03	-0.82	1.00
rhotic.low.voiced - rhotic.high.voiceless	0.14	1.11	0.12	1.00
stop.low.voiced - stop.high.voiceless	0.11	0.73	0.15	1.00
stop.low.voiced - stop.high.aspirated	-1.64	0.84	-1.96	0.63
nasal.high.voiced - nasal.high.voiceless	-1.34	1.36	-0.98	0.99
stop.high.voiceless - stop.high.aspirated	-1.75	0.86	-2.02	0.58

Table 12: Parametric coefficient estimates for H1-H2 GAMM model, Northern Kmhmu'.

	Estimate	Std. Error	t value	Pr(>|t|)
(Intercept)	216.56	11.22	19.30	0.00
manner.reg.voice1	8.41	9.60	0.88	0.38
manner.reg.voice2	4.46	6.68	0.67	0.50
manner.reg.voice3	24.58	3.13	7.84	0.00
manner.reg.voice4	3.55	4.39	0.81	0.42
manner.reg.voice5	12.38	4.27	2.90	0.00
manner.reg.voice6	-2.37	2.07	-1.15	0.25
manner.reg.voice7	8.62	1.71	5.03	0.00
manner.reg.voice8	5.47	1.77	3.08	0.00

Table 13: Estimated marginal mean differences in H1-H2 over first 10% of vowel between manner/register/voicing pairs, Northern Kmhmu'.

contrast	estimate	SE	t.ratio	p.value
liquid.low.voiced - liquid.high.voiced	0.45	0.58	0.78	1.00
nasal.low.voiced - nasal.high.voiced	0.54	1.37	0.39	1.00
rhotic.low.voiced - rhotic.high.voiced	-1.01	1.88	-0.54	1.00
stop.low.voiceless - stop.high.voiceless	-0.51	0.98	-0.52	1.00
stop.low.voiceless - stop.high.aspirated	-3.28	1.15	-2.84	0.10
stop.high.voiceless - stop.high.aspirated	-2.77	1.18	-2.35	0.31

Table 14: Estimated marginal mean differences in H1-H2 at 50% of vowel between manner/register/voicing pairs, Northern Kmhmu'.

contrast	estimate	SE	t.ratio	p.value

contrast	estimate	SE	t.ratio	p.value
liquid.low.voiced - liquid.high.voiced	0.88	0.53	1.65	0.78
nasal.low.voiced - nasal.high.voiced	0.08	1.25	0.06	1.00
rhotic.low.voiced - rhotic.high.voiced	-0.23	1.50	-0.15	1.00
stop.low.voiceless - stop.high.voiceless	-0.25	0.85	-0.30	1.00
stop.low.voiceless - stop.high.aspirated	-0.57	1.00	-0.57	1.00
stop.high.voiceless - stop.high.aspirated	-0.32	1.03	-0.31	1.00

Table 15: Parametric coefficient estimates for CPP GAMM model, Eastern Kmhmu'.

	Estimate	Std. Error	t value	Pr(>|t|)
(Intercept)	23.86	0.44	53.67	0.00
manner.reg.voice1	0.42	0.36	1.15	0.25
manner.reg.voice2	0.10	0.17	0.58	0.56
manner.reg.voice3	-0.03	0.08	-0.38	0.70
manner.reg.voice4	-0.06	0.14	-0.39	0.69
manner.reg.voice5	0.23	0.08	2.79	0.01
manner.reg.voice6	0.05	0.05	0.91	0.36
manner.reg.voice7	0.04	0.06	0.67	0.50
manner.reg.voice8	0.02	0.03	0.67	0.50
manner.reg.voice9	-0.08	0.03	-2.16	0.03

Table 16: Estimated marginal mean differences in CPP over first 10% of vowel between manner/register/voicing pairs, Eastern Kmhmu'.

contrast	estimate	SE	t.ratio	p.value

contrast	estimate	SE	t.ratio	p.value
liquid.low.voiced - liquid.high.voiced	-1.18	1.91	-0.62	1.00
nasal.low.voiced - nasal.high.voiced	-1.90	1.11	-1.71	0.79
nasal.low.voiced - nasal.high.voiceless	-1.38	0.82	-1.68	0.81
rhotic.low.voiced - rhotic.high.voiceless	0.86	1.14	0.76	1.00
stop.low.voiced - stop.high.voiceless	-2.10	0.60	-3.50	0.02
stop.low.voiced - stop.high.aspirated	2.03	0.75	2.72	0.16
nasal.high.voiced - nasal.high.voiceless	0.52	1.14	0.46	1.00
stop.high.voiceless - stop.high.aspirated	4.13	0.77	5.40	0.00

Table 17: Estimated marginal mean differences in CPP at 50% of vowel between manner/register/voicing pairs, Eastern Kmhmu'.

contrast	estimate	SE	t.ratio	p.value
liquid.low.voiced - liquid.high.voiced	0.09	1.35	0.06	1.00
nasal.low.voiced - nasal.high.voiced	-0.76	0.78	-0.98	0.99
nasal.low.voiced - nasal.high.voiceless	-0.11	0.58	-0.19	1.00
rhotic.low.voiced - rhotic.high.voiceless	-0.41	0.81	-0.51	1.00
stop.low.voiced - stop.high.voiceless	-0.01	0.42	-0.03	1.00
stop.low.voiced - stop.high.aspirated	0.04	0.53	0.08	1.00
nasal.high.voiced - nasal.high.voiceless	0.66	0.80	0.82	1.00

contrast	estimate	SE	t.ratio	p.value
stop.high.voiceless - stop.high.aspirated	0.05	0.54	0.10	1.00

Table 18: Parametric coefficient estimates for CPP GAMM model, Northern Kmhmu'.

	Estimate	Std. Error	t value	Pr(>|t|)
(Intercept)	23.80	0.72	33.01	0.00
manner.reg.voice1	0.12	0.28	0.44	0.66
manner.reg.voice2	0.02	0.18	0.09	0.93
manner.reg.voice3	0.45	0.09	4.76	0.00
manner.reg.voice4	0.11	0.11	1.01	0.31
manner.reg.voice5	0.18	0.12	1.53	0.13
manner.reg.voice6	-0.08	0.06	-1.49	0.14
manner.reg.voice7	0.09	0.05	1.88	0.06
manner.reg.voice8	-0.10	0.05	-2.10	0.04

Table 19: Estimated marginal mean differences in CPP over first 10% of vowel between manner/register/voicing pairs, Northern Kmhmu'.

contrast	estimate	SE	t.ratio	p.value
liquid.low.voiced - liquid.high.voiced	-1.43	0.53	-2.71	0.14
nasal.low.voiced - nasal.high.voiced	-0.53	1.25	-0.42	1.00
rhotic.low.voiced - rhotic.high.voiced	-0.96	1.56	-0.62	1.00
stop.low.voiceless - stop.high.voiceless	-0.49	0.89	-0.55	1.00
stop.low.voiceless - stop.high.aspirated	1.87	0.98	1.91	0.61
stop.high.voiceless - stop.high.aspirated	2.36	0.99	2.37	0.30

Table 20: Estimated marginal mean differences in CPP at 50% of vowel between manner/register/voicing pairs, Northern Kmhmu'.

contrast	estimate	SE	t.ratio	p.value
liquid.low.voiced - liquid.high.voiced	-2.07	0.46	-4.48	0.00
nasal.low.voiced - nasal.high.voiced	-0.92	0.87	-1.05	0.98
rhotic.low.voiced - rhotic.high.voiced	-1.48	1.09	-1.36	0.91
stop.low.voiceless - stop.high.voiceless	-1.25	0.63	-1.99	0.55
stop.low.voiceless - stop.high.aspirated	-0.18	0.69	-0.27	1.00
stop.high.voiceless - stop.high.aspirated	1.07	0.70	1.53	0.84

Table 21: Parametric coefficient estimates for CQ_PH GAMM model, Eastern Kmhmu'.

	Estimate	Std. Error	t value	Pr(>|t|)
(Intercept)	0.53	0.01	60.92	0.00
manner.reg.voice1	-0.01	0.01	-1.14	0.25
manner.reg.voice2	0.01	0.01	0.57	0.57
manner.reg.voice3	0.00	0.00	0.14	0.89
manner.reg.voice4	-0.01	0.00	-3.73	0.00
manner.reg.voice5	-0.02	0.00	-5.71	0.00

Table 22: Estimated marginal mean differences in CQ_PH over first 10% of vowel between manner/register/voicing pairs, Eastern Kmhmu'.

contrast	estimate	SE	t.ratio	p.value

contrast	estimate	SE	t.ratio	p.value
stop.low.voiced - stop.high.voiceless	0.07	0.05	1.31	0.78
stop.low.voiced - stop.high.aspirated	0.49	0.04	11.38	0.00
stop.high.voiceless - stop.high.aspirated	0.42	0.06	6.71	0.00

Table 23: Estimated marginal mean differences in CQ_PH at 50% of vowel between manner/register/voicing pairs, Eastern Kmhmu'.

contrast	estimate	SE	t.ratio	p.value
stop.low.voiced - stop.high.voiceless	0.02	0.03	0.65	0.99
stop.low.voiced - stop.high.aspirated	-0.04	0.03	-1.18	0.85
stop.high.voiceless - stop.high.aspirated	-0.06	0.04	-1.48	0.68

Table 24: Parametric coefficient estimates for CQ_PH GAMM model, Northern Kmhmu'.

	Estimate	Std. Error	t value	Pr(>|t|)
(Intercept)	0.45	0.01	35.07	0.00
manner.reg.voice1	0.00	0.01	0.36	0.72
manner.reg.voice2	-0.00	0.01	-0.39	0.70
manner.reg.voice3	0.00	0.01	0.12	0.90
manner.reg.voice4	-0.01	0.01	-1.61	0.11
manner.reg.voice5	-0.00	0.00	-0.14	0.89
manner.reg.voice6	-0.01	0.00	-4.41	0.00

Table 25: Estimated marginal mean differences in CQ_PH over first 10% of vowel between manner/register/voicing pairs, Northern Kmhmu'.

contrast	estimate	SE	t.ratio	p.value
stop.low.voiceless - stop.high.voiceless	-0.01	0.03	-0.25	1.00
stop.low.voiceless - stop.high.aspirated	0.31	0.04	8.32	0.00
stop.high.voiceless - stop.high.aspirated	0.32	0.04	8.06	0.00

Table 26: Estimated marginal mean differences in CQ_PH at 50% of vowel between manner/register/voicing pairs, Northern Kmhmu'.

contrast	estimate	SE	t.ratio	p.value
stop.low.voiceless - stop.high.voiceless	0.02	0.03	0.71	0.99
stop.low.voiceless - stop.high.aspirated	0.04	0.03	1.35	0.83
stop.high.voiceless - stop.high.aspirated	0.02	0.03	0.64	1.00

Table 27: Parametric coefficient estimates for F1 GAMM model, Eastern Kmhmu'.

	Estimate	Std. Error	t value	Pr(>|t|)
(Intercept)	609.04	4.52	134.78	0.00
manner.reg.voice.vowel1	-0.78	8.26	-0.09	0.92
manner.reg.voice.vowel2	-10.09	6.47	-1.56	0.12
manner.reg.voice.vowel3	-1.68	4.87	-0.34	0.73
manner.reg.voice.vowel4	-2.40	3.50	-0.69	0.49
manner.reg.voice.vowel5	11.60	2.97	3.91	0.00
manner.reg.voice.vowel6	-40.06	3.98	-10.06	0.00
manner.reg.voice.vowel7	-26.30	3.41	-7.71	0.00
manner.reg.voice.vowel8	-23.31	2.23	-10.45	0.00

	Estimate	Std. Error	t value	Pr(>|t|)
manner.reg.voice.vowel9	-9.25	2.76	-3.35	0.00
manner.reg.voice.vowel10	-15.23	1.94	-7.85	0.00
manner.reg.voice.vowel11	-5.92	2.44	-2.42	0.02
manner.reg.voice.vowel12	-10.57	1.58	-6.69	0.00
manner.reg.voice.vowel13	-8.56	1.94	-4.41	0.00
manner.reg.voice.vowel14	-20.14	1.10	-18.36	0.00
manner.reg.voice.vowel15	-18.02	1.07	-16.91	0.00
manner.reg.voice.vowel16	-15.64	0.95	-16.52	0.00
manner.reg.voice.vowel17	-14.15	1.48	-9.58	0.00
manner.reg.voice.vowel18	-13.20	1.40	-9.45	0.00
manner.reg.voice.vowel19	-13.07	1.36	-9.63	0.00
manner.reg.voice.vowel20	-10.95	0.92	-11.97	0.00
manner.reg.voice.vowel21	-10.02	1.22	-8.22	0.00
manner.reg.voice.vowel22	-9.75	1.15	-8.48	0.00

Table 28: Estimated marginal mean differences in F1 over first 10% of vowel between manner/register/voicing/vowel pairs, Eastern Kmhmu'.

contrast	estimate	SE	t.ratio	p.value
sonorant.low.voiced.aa - sonorant.high.voiced.aa	54.28	36.96	1.47	1.00
stop.low.voiced.aa - stop.high.voiceless.aa	-83.11	34.11	-2.44	0.70
stop.high.voiceless.aa - stop.high.aspirated.aa	-124.55	41.02	-3.04	0.26
stop.low.voiced.EE - stop.high.voiceless.EE	-13.69	58.53	-0.23	1.00
stop.low.voiced.OO - stop.high.voiceless.OO	8.82	51.22	0.17	1.00
stop.high.voiceless.OO - stop.high.aspirated.OO	-13.69	57.93	-0.24	1.00

contrast	estimate	SE	t.ratio	p.value
stop.low.voiced.uu - stop.high.voiceless.uu	18.30	36.65	0.50	1.00
stop.high.voiceless.uu - stop.high.aspirated.uu	52.19	49.70	1.05	1.00
stop.low.voiced.ii - stop.high.voiceless.ii	-30.28	61.59	-0.49	1.00
stop.high.voiceless.ii - stop.high.aspirated.ii	55.97	59.99	0.93	1.00

Table 29: Estimated marginal mean differences in F1 at 50% of vowel between manner/register/voicing/vowel pairs, Eastern Kmhmu'.

contrast	estimate	SE	t.ratio	p.value
sonorant.low.voiced.aa - sonorant.high.voiced.aa	31.58	27.23	1.16	1.00
stop.low.voiced.aa - stop.high.voiceless.aa	27.71	24.62	1.13	1.00
stop.high.voiceless.aa - stop.high.aspirated.aa	-52.82	29.35	-1.80	0.98
stop.low.voiced.EE - stop.high.voiceless.EE	-6.05	41.81	-0.14	1.00
stop.low.voiced.OO - stop.high.voiceless.OO	6.03	36.26	0.17	1.00
stop.high.voiceless.OO - stop.high.aspirated.OO	-8.67	41.46	-0.21	1.00
stop.low.voiced.uu - stop.high.voiceless.uu	-1.41	24.99	-0.06	1.00
stop.high.voiceless.uu - stop.high.aspirated.uu	-23.07	34.68	-0.67	1.00
stop.low.voiced.ii - stop.high.voiceless.ii	-45.30	42.61	-1.06	1.00
stop.high.voiceless.ii - stop.high.aspirated.ii	36.81	42.44	0.87	1.00

Table 30: Parametric coefficient estimates for F1 GAMM model, Northern Kmhmu'.

	Estimate	Std. Error	t value	Pr(>|t|)
(Intercept)	620.80	5.20	119.44	0.00
manner.reg.voice.vowel1	13.23	11.86	1.12	0.26
manner.reg.voice.vowel2	-7.45	8.52	-0.87	0.38
manner.reg.voice.vowel3	5.68	6.36	0.89	0.37
manner.reg.voice.vowel4	9.21	6.79	1.36	0.18
manner.reg.voice.vowel5	-53.75	5.60	-9.60	0.00
manner.reg.voice.vowel6	-33.21	4.80	-6.93	0.00
manner.reg.voice.vowel7	-31.76	3.12	-10.19	0.00
manner.reg.voice.vowel8	-19.41	2.70	-7.18	0.00
manner.reg.voice.vowel9	-20.10	3.35	-5.99	0.00
manner.reg.voice.vowel10	-13.15	1.93	-6.81	0.00
manner.reg.voice.vowel11	-11.46	2.74	-4.19	0.00
manner.reg.voice.vowel12	-8.15	1.83	-4.44	0.00
manner.reg.voice.vowel13	-4.63	2.29	-2.02	0.04
manner.reg.voice.vowel14	-3.58	2.17	-1.65	0.10
manner.reg.voice.vowel15	-22.55	1.10	-20.51	0.00
manner.reg.voice.vowel16	-17.02	1.05	-16.25	0.00
manner.reg.voice.vowel17	-18.21	1.31	-13.91	0.00
manner.reg.voice.vowel18	-13.96	1.26	-11.08	0.00
manner.reg.voice.vowel19	-13.30	1.18	-11.31	0.00
manner.reg.voice.vowel20	-14.13	1.10	-12.89	0.00
manner.reg.voice.vowel21	-11.18	1.08	-10.36	0.00
manner.reg.voice.vowel22	-13.70	1.41	-9.75	0.00
manner.reg.voice.vowel23	-10.50	0.98	-10.70	0.00
manner.reg.voice.vowel24	-10.78	1.29	-8.35	0.00

Table 31: Estimated marginal mean differences in F1 over first 10% of vowel between manner/register/voicing/vowel pairs, Northern Kmhmu'.

contrast	estimate	SE	t.ratio	p.value
sonorant.low.voiced.aa - sonorant.high.voiced.aa	23.91	37.09	0.64	1.00
stop.low.voiceless.aa - stop.high.voiceless.aa	-20.44	47.67	-0.43	1.00
stop.low.voiceless.aa - stop.high.aspirated.aa	-143.37	58.84	-2.44	0.74
stop.high.voiceless.aa - stop.high.aspirated.aa	-122.93	59.18	-2.08	0.93
sonorant.low.voiced.ee - sonorant.high.voiced.ee	-69.27	66.87	-1.04	1.00
stop.low.voiceless.ee - stop.high.voiceless.ee	-42.50	47.33	-0.90	1.00
stop.high.voiceless.ee - stop.high.aspirated.ee	29.90	58.14	0.51	1.00
sonorant.low.voiced.oo - sonorant.high.voiced.oo	12.48	54.97	0.23	1.00
stop.low.voiceless.oo - stop.high.voiceless.oo	7.46	57.24	0.13	1.00
stop.high.voiceless.oo - stop.high.aspirated.oo	-18.09	66.15	-0.27	1.00
sonorant.low.voiced.uu - sonorant.high.voiced.uu	-46.31	13.84	-3.35	0.13
stop.low.voiceless.uu - stop.high.voiceless.uu	-40.05	43.28	-0.93	1.00
stop.high.voiceless.uu - stop.high.aspirated.uu	31.07	46.23	0.67	1.00
sonorant.low.voiced.ii - sonorant.high.voiced.ii	-39.22	45.36	-0.86	1.00
stop.low.voiceless.ii - stop.high.voiceless.ii	-64.56	55.32	-1.17	1.00
stop.high.voiceless.ii - stop.high.aspirated.ii	57.98	54.70	1.06	1.00

Table 32: Estimated marginal mean differences in F1 at 50% of vowel between manner/register/voicing/vowel pairs, Northern Kmhmu'.

contrast	estimate	SE	t.ratio	p.value
sonorant.low.voiced.aa - sonorant.high.voiced.aa	-25.20	28.42	-0.89	1.00
stop.low.voiceless.aa - stop.high.voiceless.aa	-27.75	37.46	-0.74	1.00
stop.low.voiceless.aa - stop.high.aspirated.aa	-35.20	45.91	-0.77	1.00
stop.high.voiceless.aa - stop.high.aspirated.aa	-7.46	46.24	-0.16	1.00
sonorant.low.voiced.ee - sonorant.high.voiced.ee	-72.96	52.55	-1.39	1.00
stop.low.voiceless.ee - stop.high.voiceless.ee	-65.47	37.18	-1.76	0.99
stop.high.voiceless.ee - stop.high.aspirated.ee	71.07	45.72	1.55	1.00
sonorant.low.voiced.oo - sonorant.high.voiced.oo	-4.13	43.17	-0.10	1.00
stop.low.voiceless.oo - stop.high.voiceless.oo	-44.69	44.87	-1.00	1.00
stop.high.voiceless.oo - stop.high.aspirated.oo	-3.78	51.73	-0.07	1.00
sonorant.low.voiced.uu - sonorant.high.voiced.uu	-46.21	11.78	-3.92	0.02
stop.low.voiceless.uu - stop.high.voiceless.uu	-31.07	35.05	-0.89	1.00
stop.high.voiceless.uu - stop.high.aspirated.uu	-0.15	36.07	-0.00	1.00
sonorant.low.voiced.ii - sonorant.high.voiced.ii	-33.53	35.61	-0.94	1.00
stop.low.voiceless.ii - stop.high.voiceless.ii	-39.75	43.12	-0.92	1.00
stop.high.voiceless.ii - stop.high.aspirated.ii	11.71	42.72	0.27	1.00

Table 33: Parametric coefficient estimates for F2 GAMM model, Eastern Kmhmu'.

	Estimate	Std. Error	t value	Pr(>|t|)
(Intercept)	1,623.64	18.98	85.55	0.00
manner.reg.voice.vowel1	10.86	32.25	0.34	0.74
manner.reg.voice.vowel2	5.69	24.41	0.23	0.82
manner.reg.voice.vowel3	10.64	16.07	0.66	0.51
manner.reg.voice.vowel4	1.50	14.29	0.11	0.92
manner.reg.voice.vowel5	-2.80	11.80	-0.24	0.81
manner.reg.voice.vowel6	57.95	16.65	3.48	0.00
manner.reg.voice.vowel7	58.30	14.52	4.02	0.00
manner.reg.voice.vowel8	38.61	9.50	4.06	0.00
manner.reg.voice.vowel9	-62.59	11.50	-5.44	0.00
manner.reg.voice.vowel10	-55.81	7.60	-7.35	0.00
manner.reg.voice.vowel11	-42.79	9.60	-4.46	0.00
manner.reg.voice.vowel12	-41.14	6.40	-6.42	0.00
manner.reg.voice.vowel13	-38.76	8.17	-4.75	0.00
manner.reg.voice.vowel14	-44.30	4.66	-9.51	0.00
manner.reg.voice.vowel15	-39.86	4.35	-9.16	0.00
manner.reg.voice.vowel16	-37.51	4.04	-9.29	0.00
manner.reg.voice.vowel17	-32.90	6.33	-5.20	0.00
manner.reg.voice.vowel18	45.32	6.10	7.43	0.00
manner.reg.voice.vowel19	54.89	5.86	9.37	0.00
manner.reg.voice.vowel20	41.66	4.16	10.01	0.00
manner.reg.voice.vowel21	37.63	5.60	6.71	0.00
manner.reg.voice.vowel22	41.07	5.07	8.10	0.00

Table 34: Estimated marginal mean differences in F2 over first 10% of vowel between manner/register/voicing/vowel pairs, Eastern Kmhmu'.

contrast	estimate	SE	t.ratio	p.value
sonorant.low.voiced.aa - sonorant.high.voiced.aa	-55.06	93.53	-0.59	1.00
stop.low.voiced.aa - stop.high.voiceless.aa	85.14	91.31	0.93	1.00
stop.high.voiceless.aa - stop.high.aspirated.aa	-175.90	109.15	-1.61	0.99
stop.low.voiced.EE - stop.high.voiceless.EE	-95.70	161.56	-0.59	1.00
stop.low.voiced.OO - stop.high.voiceless.OO	297.92	129.55	2.30	0.80
stop.high.voiceless.OO - stop.high.aspirated.OO	49.95	149.81	0.33	1.00
stop.low.voiced.uu - stop.high.voiceless.uu	162.59	110.95	1.47	1.00
stop.high.voiceless.uu - stop.high.aspirated.uu	99.03	150.28	0.66	1.00
stop.low.voiced.ii - stop.high.voiceless.ii	345.46	195.71	1.77	0.98
stop.high.voiceless.ii - stop.high.aspirated.ii	-352.45	192.29	-1.83	0.98

Table 35: Estimated marginal mean differences in F2 at 50% of vowel between manner/register/voicing/vowel pairs, Eastern Kmhmu'.

contrast	estimate	SE	t.ratio	p.value
sonorant.low.voiced.aa - sonorant.high.voiced.aa	-21.22	84.22	-0.25	1.00
stop.low.voiced.aa - stop.high.voiceless.aa	-11.95	82.22	-0.15	1.00
stop.high.voiceless.aa - stop.high.aspirated.aa	46.89	97.15	0.48	1.00
stop.low.voiced.EE - stop.high.voiceless.EE	-54.13	145.09	-0.37	1.00

contrast	estimate	SE	t.ratio	p.value
stop.low.voiced.OO - stop.high.voiceless.OO	28.42	116.59	0.24	1.00
stop.high.voiceless.OO - stop.high.aspirated.OO	-7.87	138.91	-0.06	1.00
stop.low.voiced.uu - stop.high.voiceless.uu	63.81	98.42	0.65	1.00
stop.high.voiceless.uu - stop.high.aspirated.uu	-34.37	135.05	-0.25	1.00
stop.low.voiced.ii - stop.high.voiceless.ii	191.32	175.36	1.09	1.00
stop.high.voiceless.ii - stop.high.aspirated.ii	-177.40	173.48	-1.02	1.00

Table 36: Parametric coefficient estimates for F2 GAMM model, Northern Kmhmu'.

	Estimate	Std. Error	t value	Pr(>|t|)
(Intercept)	1,638.10	16.31	100.43	0.00
manner.reg.voice.vowel1	-0.35	37.21	-0.01	0.99
manner.reg.voice.vowel2	-5.41	26.40	-0.20	0.84
manner.reg.voice.vowel3	-10.75	19.45	-0.55	0.58
manner.reg.voice.vowel4	-22.34	20.51	-1.09	0.28
manner.reg.voice.vowel5	73.34	17.15	4.28	0.00
manner.reg.voice.vowel6	35.97	15.17	2.37	0.02
manner.reg.voice.vowel7	42.47	9.98	4.25	0.00
manner.reg.voice.vowel8	27.40	8.43	3.25	0.00
manner.reg.voice.vowel9	33.92	10.17	3.33	0.00
manner.reg.voice.vowel10	-71.66	5.58	-12.85	0.00
manner.reg.voice.vowel11	-40.38	8.36	-4.83	0.00
manner.reg.voice.vowel12	-51.90	5.55	-9.35	0.00
manner.reg.voice.vowel13	-40.27	6.96	-5.79	0.00

	Estimate	Std. Error	t value	Pr(>|t|)
manner.reg.voice.vowel14	-38.64	6.53	-5.92	0.00
manner.reg.voice.vowel15	-46.96	3.27	-14.35	0.00
manner.reg.voice.vowel16	-46.35	3.16	-14.67	0.00
manner.reg.voice.vowel17	-39.61	3.90	-10.15	0.00
manner.reg.voice.vowel18	-31.69	3.68	-8.60	0.00
manner.reg.voice.vowel19	-30.62	3.49	-8.76	0.00
manner.reg.voice.vowel20	51.03	3.42	14.93	0.00
manner.reg.voice.vowel21	39.62	3.56	11.14	0.00
manner.reg.voice.vowel22	40.14	4.53	8.86	0.00
manner.reg.voice.vowel23	32.20	3.72	8.65	0.00
manner.reg.voice.vowel24	27.09	4.75	5.70	0.00

Table 37: Estimated marginal mean differences in F2 over first 10% of vowel between manner/register/voicing/vowel pairs, Northern Kmhmu'.

contrast	estimate	SE	t.ratio	p.value
sonorant.low.voiced.aa - sonorant.high.voiced.aa	-79.12	90.92	-0.87	1.00
stop.low.voiceless.aa - stop.high.voiceless.aa	-3.65	113.84	-0.03	1.00
stop.low.voiceless.aa - stop.high.aspirated.aa	78.02	139.45	0.56	1.00
stop.high.voiceless.aa - stop.high.aspirated.aa	81.67	141.22	0.58	1.00
sonorant.low.voiced.ee - sonorant.high.voiced.ee	138.84	189.13	0.73	1.00
stop.low.voiceless.ee - stop.high.voiceless.ee	82.63	131.36	0.63	1.00
stop.high.voiceless.ee - stop.high.aspirated.ee	-188.68	150.97	-1.25	1.00

contrast	estimate	SE	t.ratio	p.value
sonorant.low.voiced.oo - sonorant.high.voiced.oo	-117.85	144.61	-0.81	1.00
stop.low.voiceless.oo - stop.high.voiceless.oo	72.36	128.94	0.56	1.00
stop.high.voiceless.oo - stop.high.aspirated.oo	80.29	143.72	0.56	1.00
sonorant.low.voiced.uu - sonorant.high.voiced.uu	-28.35	43.69	-0.65	1.00
stop.low.voiceless.uu - stop.high.voiceless.uu	35.32	120.73	0.29	1.00
stop.high.voiceless.uu - stop.high.aspirated.uu	91.75	121.85	0.75	1.00
sonorant.low.voiced.ii - sonorant.high.voiced.ii	140.27	153.12	0.92	1.00
stop.low.voiceless.ii - stop.high.voiceless.ii	203.26	172.59	1.18	1.00
stop.high.voiceless.ii - stop.high.aspirated.ii	-58.62	179.39	-0.33	1.00

Table 38: Estimated marginal mean differences in F2 at 50% of vowel between manner/register/voicing/vowel pairs, Northern Kmhmu'.

contrast	estimate	SE	t.ratio	p.value
sonorant.low.voiced.aa - sonorant.high.voiced.aa	-18.67	77.98	-0.24	1.00
stop.low.voiceless.aa - stop.high.voiceless.aa	21.94	100.90	0.22	1.00
stop.low.voiceless.aa - stop.high.aspirated.aa	91.49	122.84	0.74	1.00
stop.high.voiceless.aa - stop.high.aspirated.aa	69.55	123.34	0.56	1.00
sonorant.low.voiced.ee - sonorant.high.voiced.ee	182.93	153.43	1.19	1.00
stop.low.voiceless.ee - stop.high.voiceless.ee	66.70	109.75	0.61	1.00

contrast	estimate	SE	t.ratio	p.value
stop.high.voiceless.ee - stop.high.aspirated.ee	-120.98	127.01	-0.95	1.00
sonorant.low.voiced.oo - sonorant.high.voiced.oo	-185.41	121.51	-1.53	1.00
stop.low.voiceless.oo - stop.high.voiceless.oo	-91.73	118.00	-0.78	1.00
stop.high.voiceless.oo - stop.high.aspirated.oo	58.82	134.84	0.44	1.00
sonorant.low.voiced.uu - sonorant.high.voiced.uu	0.40	32.87	0.01	1.00
stop.low.voiceless.uu - stop.high.voiceless.uu	-11.65	102.15	-0.11	1.00
stop.high.voiceless.uu - stop.high.aspirated.uu	19.24	102.94	0.19	1.00
sonorant.low.voiced.ii - sonorant.high.voiced.ii	123.63	117.91	1.05	1.00
stop.low.voiceless.ii - stop.high.voiceless.ii	129.67	144.49	0.90	1.00
stop.high.voiceless.ii - stop.high.aspirated.ii	67.76	152.65	0.44	1.00
